# Proteomic Insight into the Role of Exosomes in Proliferative Vitreoretinopathy Development

**DOI:** 10.3390/jcm11102716

**Published:** 2022-05-11

**Authors:** Gopa Kumar Gopinadhan Nair, Dimitrios Pollalis, Jonathan D. Wren, Constantin Georgescu, Virginie Sjoelund, Sun Young Lee

**Affiliations:** 1 Ophthalmology, Dean McGee Eye Institute, University of Oklahoma Health Sciences Center, Oklahoma City, OK 73104, USA; gopakumar-gopinadhannair@ouhsc.edu; 2 USC Roski Eye Institute, USC Ginsburg Institute for Biomedical Therapeutics and Department of Ophthalmology, Keck School of Medicine, University of Southern California, 1450 San Pablo, Los Angeles, CA 90033, USA; pollalis@usc.edu; 3 Genes & Human Diseases Research Program, Oklahoma Medical Research Foundation, Oklahoma City, OK 73104, USA; jonathan-wren@omrf.org (J.D.W.); constantin-georgescu@omrf.org (C.G.); 4 Department of Cell Biology, University of Oklahoma Health Sciences Center, Oklahoma City, OK 73104, USA; virginie-sjoelund@ouhsc.edu; 5 Department of Physiology, University of Oklahoma Health Sciences Center, Oklahoma City, OK 73104, USA

**Keywords:** exosome, proteome, PVR

## Abstract

**Purpose**: To characterize vitreous humor (VH) exosomes and to explore their role in the development of proliferative vitreoretinopathy (PVR) using mass spectrometry-based proteome profiling. **Methods**: Exosomes were isolated from undiluted VH from patients with retinal detachment (RD) with various stages of PVR (*n* = 9), macular hole (MH; *n* = 5), or epiretinal membrane (ERM; *n* = 5) using differential ultracentrifugation. The exosomal size, morphology, and exosome markers were analyzed using a nanoparticle tracking analysis (NTA), transmission electron microscopy (TEM), and an exosome detection antibody array. The tryptic fragment sequencing of exosome-contained proteins was performed using liquid chromatography–tandem mass spectrometry (LC-MS/MS) and a Thermo Lumos Fusion Tribrid Orbitrap mass spectrometer. The pathway analysis of the MS data was performed. **Results**: The number of exosome particles were significantly increased only in the RD with severe PVR group compared with the control groups and the RD without PVR or with mild PVR groups. Of 724 exosome proteins identified, 382 were differentially expressed (DE) and 176 were uniquely present in PVR. Both DE proteins and exosome proteins that were only present in PVR were enriched in proteins associated with previously known key pathways related to PVR development, including reactive retinal gliosis, pathologic cellular proliferation, inflammation, growth of connective tissues, and epithelial mesenchymal transition (EMT). The SPP1, CLU, VCAN, COL2A1, and SEMA7A that are significantly upregulated in PVR were related to the tissue remodeling. **Conclusions**: Exosomes may play a key role in mediating tissue remodeling along with a complex set of pathways involved in PVR development.

## 1. Introduction

**Figure 1:**
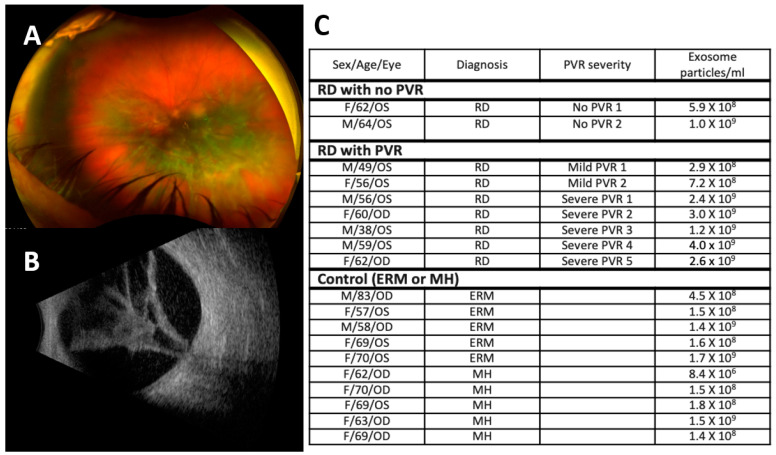
Clinical findings in severe proliferative vitreoretinopathy (PVR) and patient demographics. (**A**) Fundus image and (**B**) B-scan of severe PVR demonstrating vitreous opacity, star folds, and fibrous proliferation that led to closed tractional retinal detachment. (**C**) Patient demographics. RD, retinal detachment; PVR, proliferative vitreoretinopathy; ERM, epiretinal membrane; MH, macular hole.

Proliferative vitreoretinopathy (PVR) is clinically characterized by vitreous opacity, retinal wrinkles, or stiffness in its early stage and fibrous membrane formation in endovitreous, subretinal, and epiretinal membranes that exerts traction on the retinal surface in its advanced stage (Figure 1A,B) [1,2,3]. PVR can occur in various pathologies, such as retinal detachment and ocular trauma, or as an inadvertent complication of retinal surgery, such as stem cell injection [1,2,3]. Extensive laboratory research, including histopathologic studies of the human PVR tissues, revealed that PVR involves a complex disease pathway, including reactive retinal gliosis, activation of fibroblasts and inflammatory cells, and excessive deposition of the extracellular matrix (ECM), similar to the wound-healing process (retina keloid) [4,5,6,7,8,9,10]. Multiple cellular components involving retinal glial cells, including Müller cells, astrocytes and microglia, retinal pigment epithelial cells (RPE), hyalocytes, and inflammatory cells, including macrophages, lymphocytes, neutrophils, fibrocytes, and myofibrocytes, have been recognized during PVR development [4,5,6,7,8,9,10]. Despite considerable research that consistently demonstrated that PVR involves a complex disease pathway and multiple cellular involvements, the clinical management of PVR has not improved over the last 30 years. PVR remains a leading cause of surgical failure in vitreoretinal surgeries. How a complex pathway in PVR is orchestrated during the disease process is not completely understood.

The failure of several attempted clinical trials with antiproliferative agents, including 5-fluouracil and daunorubicin, and an anti-inflammatory agent (steroid) further suggests that targeting a single pathway may not be sufficient to tackle PVR [11,12,13,14].

We recently reported that the levels of glial fibrillary acid protein (GFAP) and vimentin, hallmarks of retinal gliosis, were increased more than 30-fold in the VH from PVR patients compared with patients with macular hole (MH) or epiretinal membrane (ERM), and these levels were highly correlated with the clinical severity of PVR [15]. Because reactive gliosis has been reported as a nonspecific reaction to many other types of retinal stress, it has been speculated that reactive gliosis may be a minor player in PVR formation. Nevertheless, how reactive gliosis can be correlated to the severity of PVR remains poorly understood. Exosomes could be an important variable in this equation. Yet, to our knowledge, no studies have characterized the exosomal content in PVR.

Exosomes make up the smallest-sized subset (diameter ≈ 30–150 nm) of extracellular vesicles (EVs) (diameter ≈ 30–1000 nm) that are lipid bilayer-delimited nanovesicles naturally released from a cell [16,17,18]. Recent studies revealed that exosomes selectively incorporate proteins, lipids, and non-coding RNA from their host cell, and the exosomal contents can be transferred to target recipient cells, playing a key role in cell-to-cell communication [16,17,18]. Despite recently increased efforts to characterize the protein content of exosomes from different types of biological fluids, exosomes released to vitreous humor (VH) have been limitedly characterized, and their protein composition and pathologic role in PVR have not been studied [19,20,21,22,23,24,25].

Capitalizing on the known pathways of PVR diseases, the emerging roles of exosomes in other pathologic diseases, and our recent study demonstrating that retinal gliosis is associated with the severity of PVR, we hypothesized that exosomes released into VH play a role in PVR development by mediating the complex cellular and diseases processes involved in PVR. Therefore, the present study aimed to characterize VH exosomes and to discover their role, focused on the known multiple pathologic disease pathways involved in PVR by proteomic profiles of VH exosomes in patients with PVR.

## 2. Material and Methods

### 2.1. Patients

Nineteen patients who underwent pars plana vitrectomy for retinal detachment (RD) with various degrees of proliferative vitreoretinopathy (RD; *n* = 9), idiopathic full-thickness macular hole (MH; *n* = 5), or epiretinal membrane (ERM; *n* = 5) were included. All patients underwent comprehensive eye exams that included a slit-lamp examination and dilated fundus exam. PVR severity was classified as mild (Grade A or B) or severe (Grade C or D) using PVR classification (The Retina Society Terminology Committee, 1983) based on pre-operative and intra-operative clinical findings [14].

### 2.2. Vitreous Humor (VH) Preparation

Undiluted vitreous humor (VH) (0.5–1.0 mL volume) was obtained during the vitrectomy procedure and was processed and stored as previously described [15].

### 2.3. Exosome Isolation and Characterization

Exosomes were purified using differential ultracentrifugation. VH was thawed and mixed in an ultra-clear centrifuge tube (Beckman Coulter No. 344062) with 1.5 mL of phosphate-buffered saline (pH7.4) containing a proteinase inhibitor cocktail. Samples were spun at 2000× *g* at 4 °C for 30 min and the supernatant was collected. The supernatant was again subjected to ultracentrifugation (Beckman Coulter Optima^TM^ L-80 XP Ultracentrifuge with rotor SW60 Ti, 4 mL × 6) at 12,000× *g* at 4 °C for 60 min and the resulted supernatant was collected in a new ultra-clear centrifuge tube and spun at 120,000× *g* for 120 min at 4 °C. The final pellet (exosomes) was resuspended in 100 μL PBS, pH 7.4, and stored at −80 °C until analysis.

The size, number, and morphology of exosomes were determined by nanoparticle tracking analysis (NTA) (NanoSight, Malvern Instruments Ltd., Malvern, UK) and transmission electron microscopy (TEM). Exosome markers were analyzed by exosome detection antibody array (Exo-Check™, System Biosciences, Palo Alto, CA, USA) per the manufacturer’s instruction.

### 2.4. Proteomic Analysis

Total exosome protein concentration was determined using bicinchoninic acid assay (BCA) reagent (Abcam; Cambridge, United Kingdom) as per the manufacturer’s instructions, as previously described [22]. A total of 50 μg of exosome protein from each sample was digested with 10 µg Promega Sequencing Grade Modified Trypsin (Promega V5111, using the manufacturer’s protocols) for overnight incubation at 37 °C in 40 mM NH_4_HCO_3_. The tryptic peptides were then desalted and concentrated using Pierce™ C18 spin columns (Thermo Fischer Scientific, Waltham, WA, USA) to remove large undigested fragments and clean salt contaminants from the tryptic fragments. A total of 1 μg of tryptic peptides was then loaded onto a C18 sequencing column (Acclaim^TM^ PepMap^TM^ 100C18, ThermoFisher, Waltham, MA, USA) and eluted using a 90 min acetonitrile gradient. Eluted fragments were analyzed by LC-MS/MS analysis using a Thermo Lumos Fusion Tribrid Orbitrap mass spectrometer coupled to an Ultimate 3000 RSLC nano ultra-high-performance liquid chromatography (UHPLC). Protein identification utilized the SEQUEST search engine and the human Uniprot reference proteome database version 20201123 (42,412 reviewed and 54,052 unreviewed). Protein identification required detection of at least two peptides per protein. Database search parameters were restricted to three missed tryptic cleavage sites, a precursor ion mass tolerance of 10 ppm, a fragment ion mass tolerance of 0.03 Da, and a false discovery rate of ≤1%. Fixed protein modification was Cys carboxymethylation (+58 Da). Variable protein modifications included Met oxidation (+16 Da) and N-terminal acetylation (+42). Reporting of gene products/proteins followed the general guidelines recommended by the human eye proteome project [26]. In brief, three biological replicates were used, and the search parameters in Proteomic Discoverer 2.4 9Thermo) were set to: ≥7 amino acids minimum peptide length; ≥2 peptide matches; and protein FDR cutoff ≥1%. MS spectral files are provided in the supplementary information.

## 3. Data and Bioinformatics Analysis

Peptide count normalization and differential analysis were performed using the DESeq2 package from Bioconductor. Five pairs of sample groups, representing the progressive stages of the disease, were compared to identify proteins significantly changed. The *p*-values returned by the DESeq testing procedure were adjusted for multiple testing using the false discovery rate (FDR). FDR ≤ 0.05 was used as threshold of significance to select differentially expressed proteins.

We also searched a collection of 608 papers that mention at least 100 gene names according to NCBI’s curated mapping of Entrez Gene IDs to PubMed IDs (PMIDs), as given in their FTP-downloadable file (ftp://ftp.ncbi.nlm.nih.gov/gene/DATA/gene2pubmed.gz; accessed on 1 October 2021). Bonferroni-corrected Chi-square tests were used to estimate significance of protein overlap [27]. Ingenuity pathway analysis (IPA), focused on overlap with the known PVR disease pathways.

## 4. Results

### 4.1. Characterization of Exosomes Isolated from Vitreous Humor (VH)

**Figure 2:**
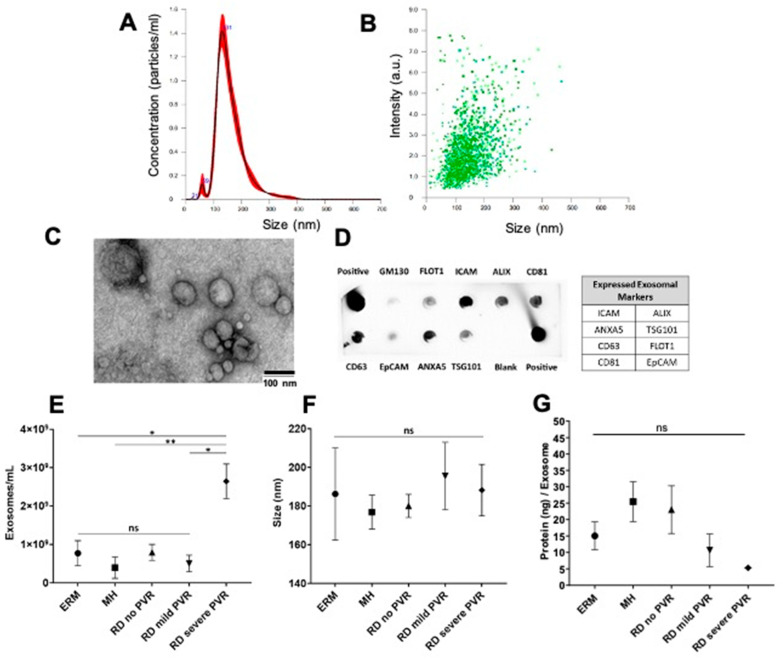
Characterization of exosomes from vitreous humor (VH). (**A**,**B**) The average peak size of exosome particles (131 nm) and average number of particles of exosome (2.42 × 10^9^/mL) were determined by nanoparticle tracking analysis (NAT). (**C**) The morphology of exosomes was confirmed by transmission electron microscopy (TEM). (**D**) Exosome markers including FLOT1, ICAM, ALIX, EpCAM, ANXA5, TSG101, CD81, and CD63 were confirmed by exosome detection antibody array. (**E**–**G**) The number of exosome particles were increased only in the RD with severe PVR group compared with the control and RD without or with mild PVR groups. The sizes of exosomes and the protein concentrations of the exosomes were similar amongst the groups (mean ± SEM; *, *p* < 0.05; **, *p* < 0.01).

The demographic characteristics of the patients and the description of the corresponding VH exosomes are summarized in Figure 1. The study groups consisted of a total of 19 patients. The control group included five ERM patients and five MH patients. The RD group included nine patients with two no PVR patients, two mild PVR patients, and five severe PVR patients. We recovered a range of 0.08–40.0 × 10^8^ exosome particles per ml vitreous sample (average 7.72 × 10^8^ particles/mL in ERM, 3.96 × 10^8^ particles/mL in MH, and 10.76 × 10^8^ in RD with/without PVR). We confirmed that our samples were enriched in exosomes by their size (peak ~131 nm), morphology, and positive exosome markers, including FLOT1, ICAM, ALIX, EpCAM, ANXA5, TSG101, CD81, and CD63 (Figure 2A–D). The number of exosome particles were significantly increased only in the RD with severe PVR group compared with the control groups and the RD without PVR or with mild PVR groups. Neither the size of the exosomes nor the protein concentration of the exosomes was significantly different among the groups. (Figure 2E–G).

### 4.2. Proteins Detected in Exosomes with PVR and Differential Protein Expression

**Figure 3:**
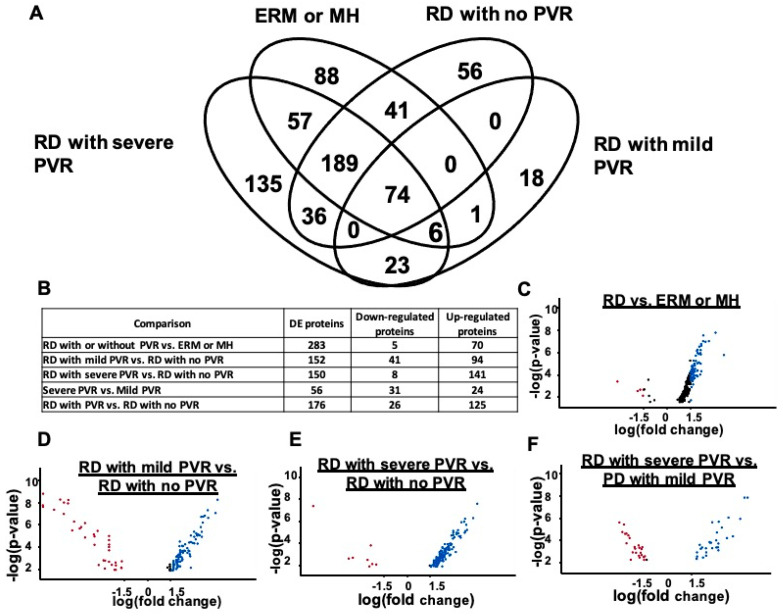
Proteomic analysis of exosome proteins from vitreous humor with PVR. (**A**) Venn diagram representing the total number of proteins identified (*n* = 724). (**B**) Differentially expressed (DE) proteins in table and (**C**–**F**) in volcano plots with upregulated (blue dots) and downregulated (red dots) proteins.

The LC-MS/MS analysis of the vitreous exosomes from the RD with/without PVR and control (MH and ERM) groups identified 724 proteins. Of these, 263 proteins were shared among groups. The abundant shared proteins included ALB, TF, C3, SERPINA1, IGHG1, CLU, APOA1, IGKC, and C4. Of the 724 proteins, 176 proteins were uniquely present in PVR, and 135 proteins were only present in severe PVR (Figure 3A). From the literature-based pathway analysis, the top seven most significant overlaps detected were all *p* < 0.01, with a study of G-glycosylated plasma proteins as the top hit [27,28]. Five of the remaining six top hits were prior exosome studies [29,30,31,32,33]. This confirms exosomal enrichment in our experiment and strongly suggests that the changes in protein N-glycosylation were enriched in N-glycosylation (data not shown).

We determined which of the 724 proteins were significantly differentially expressed (DE) at *fdr* < 0.05 and identified 382 DE exosome proteins between at least two conditions. The number of the DE exosomal proteins observed between conditions were as follows: (ERM and MH) vs. RD, 283; RD with no PVR vs. RD with mild PVR, 152; RD with no PVR vs. RD with severe PVR, 150; RD with mild PVR vs. RD with severe PVR, 56; and RD with no PVR vs. RD with PVR regardless of severity, 176. The number of the DE proteins are displayed in Figure 3B and in the volcano plots demonstrating upregulated and downregulated proteins between different groups (*fdr* < 0.05; Figure 3C–F). Among the significantly upregulated proteins, C4B, DKK3, CST3, VCAN, QSOX1, CLSTN1, FBN1, HSPG2, APLP1, and CLU were upregulated in RD with PVR compared with the control (ERM and MH). DKK3, CST3, APLP2, SPP1, CLSTN1, SEMA7A, IGLL5, FSTL5, COL2A1, and HSPG2 were upregulated in RD with PVR compared with RD with no PVR. More detailed information can be found in the Supplement.

### 4.3. Stage-Specific Protein Expression

**Figure 4:**
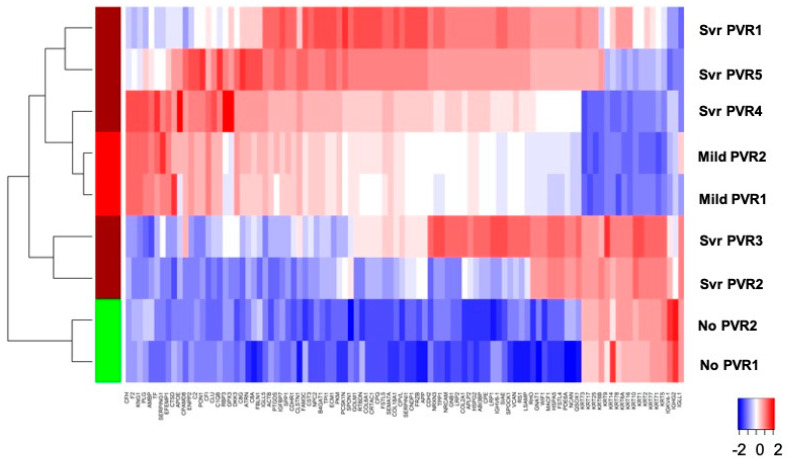
Differentially expressed proteins among different stages of PVR represented by hierachical clustering. A total of 176 proteins were differentially expressed between RD with no PVR, mild PVR, and severe PVR. Upregulated and downregulated proteins were determined (*fdr* < 0.05). Results for top 98 proteins (*fdr* < 0.01) are presented as heatmap and display protein expression levels on a logarithmic scale, rescaled to z-scores by protein. Original expression values and clustering membership details, for the 98 proteins represented in the heatmap, are listed in the supplement Table S3. The values in the heat map are normalized by gene, which fits from −2 to 2 range.

To characterize the different clinical stages of no PVR, mild PVR, and severe PVR with DE exosome proteins, the intensities of 179 DE exosome proteins in PVR were analyzed by hierarchical clustering. The results are represented as a heatmap (upregulated and downregulated; *fdr* < 0.05). An overlap between mild PVR and severe PVR was observed. However, there was a distinct difference in RD with no PVR vs. RD with PVR (Figure 4).

### 4.4. Pathway Analysis

**Figure 5:**
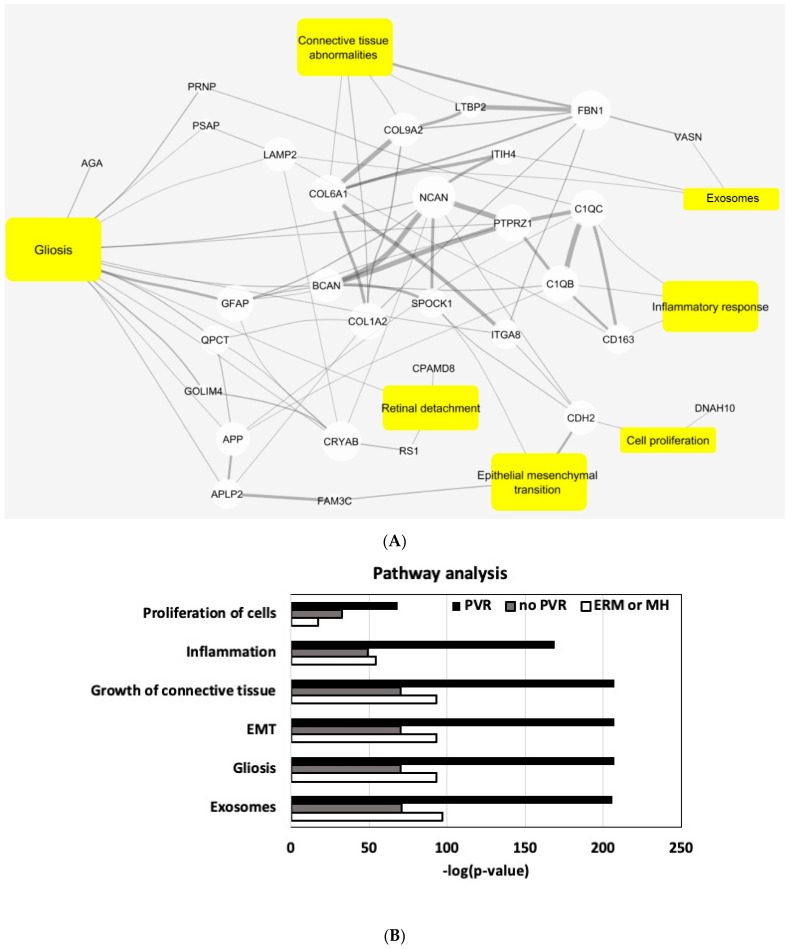

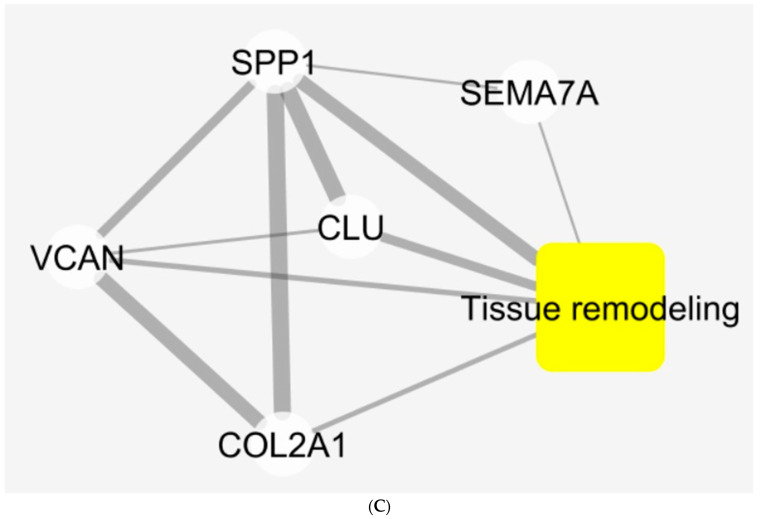
Literature and pathway analysis. (**A**) A total of 382 differentially expressed (DE) proteins were analyzed using a literature analysis with a focus on PVR diseases pathways. Node size correlates with number of connections. Line width correlates with mutual information between nodes. (**B**) A total of 268 proteins were uniquely present in the PVR groups compared with RD with no PVR, and ERM or MH groups were analyzed by ingenuity pathway analysis (IPA) with a focus on PVR diseases pathways. (**C**). From the literature analysis, SPP1, CLU, VCAN, COL2A1, and SEMA7A that are significantly upregulated in PVR were related to tissue remodeling.

To identify global biological processes potentially disturbed by PVR, we performed an enrichment analysis of the DE proteins on known functional protein sets, using a literature-based text-mining and ingenuity pathway analysis (IPA), focusing on the overlap with the known PVR disease pathways [27]. In the literature-based analysis, using mutual information to normalize term frequency, we observed a number of our DE proteins that overlapped with PVR-related concepts in PubMed Abstracts. These concepts include gliosis, connective tissue abnormalities, cell proliferation, inflammatory response, and exosomes. The previous literature reported fifteen DE proteins that overlapped in severe/mild PVR and controls and were associated with gliosis (Figure 5A).

For a more specific analysis, we analyzed a group of proteins that are uniquely present in each group (control vs. RD with no PVR vs. RD with PVR). We highlighted known key PVR pathways using the IPA to classify 268 proteins that are uniquely present in RD compared with the control group. All key PVR pathways, including the cell proliferation, inflammation, growth of connective tissues, and gliosis, were overrepresented in the RD with PVR compared with the RD with no PVR and ERM/MH groups (Figure 5B). A further literature analysis identified that the SPP1, CLU, VCAN, COL2A1, and SEMA7A that are significantly upregulated in PVR were related to tissue remodeling (Figure 5C).

## 5. Discussion

Numerous previous studies have demonstrated that PVR is a complex cellular reaction representing a vitreoretinal wound-healing response, resulting in poor visual outcomes with few therapeutic options [4,5,6,7,8,9,10]. Despite the successful discovery of PVR disease pathways, such as reactive retinal gliosis, inflammatory process, cellular proliferation, and the modification of ECM, it has not been fully understood how these pathways involving multiple cells were coordinated during PVR development. In the present study, we conducted a proteomic analysis of exosomes isolated from VH to study the role of exosomes during PVR development. We demonstrated that the number of exosome particles in VH were increased in the RD with severe PVR group compared with the control and RD without or RD with mild PVR groups. The particle size and protein amount from exosomes had some variances; however, they were not significantly different among our groups. Our result suggests that increased genesis and/or secretion of exosome particles into VH can be associated with the development of pathologic ocular conditions, such as severe PVR. Although many aspects of the biology of exosomes in disease development remain to be explored, our data indicate that strategies that interfere with the genesis and/or secretion of exosomes and impair exosome-mediated cell-to-cell communication could potentially be a therapeutic approach for PVR.

The proteomic analysis of VH exosomes using the LC-MS/MS identified PVR-unique exosomal protein expressions that are only present in PVR and differentially expressed in different stages of PVR (RD with no PVR, RD with mild PVR, and RD with severe PVR), suggesting that exosome proteins are involved in the development of PVR. An overlapped clustering between mild and severe PVR may be explained by clinical observations in PVR for which the current grading system for PVR may not be directly reflective of the stages of PVR. For example, vitreous opacity in Grade A does not always present when there is a retinal fold in Grade C. Therefore, it is possible that the clinical grading system may not directly correlate with proteomic changes, which could explain an overlapped clustering. Nevertheless, we observed a distinct difference between absent PVR and present PVR with an overlapping during the development of the spectrum of mild and severe PVR, which would provide further insights on the heterogenous clinical findings and proteomic changes of PVR.

VH, unlike other body fluids, reflects molecular signaling of the retina, particularly in pathologic conditions, because the metabolic exchange between systemic circulation and VH is relatively slow [34,35,36,37,38]. Distinctive protein profiles from VH in vasoproliferative ocular conditions may also be affected by a disrupted blood–retinal barrier, detached retina, and migrating RPE. Therefore, several studies that carried out a proteomic analysis of VH proteins from PVR concluded that PVR is a complicated pathological process with numerous proteins involved in metabolism dysfunction, immune and inflammatory reactions, and cytoskeleton remodeling [34,35,36]. However, VH proteins contain a mixture of proteins that are exosome-associated and other soluble and non-soluble proteins from various origins. A proteomic analysis of VH exosomes in PVR has not been conducted. Exosomes in VH represent a unique compartment of proteins because of their release from the cells in a controlled fashion. For example, previous proteomic studies on human PVR identified candidate protein biomarkers and pathways, including Kinogen-1, mTOR, and EFEMP-1. Kinongen-1 and EFEMP-1 were present in exosome proteins from ERM, MH, and more frequently in PVR. However, mTOR was not identified in any cohort, which further confirms that exosomes in VH represent a unique compartment of proteins, while VH proteins contain a mixture of proteins including exosome-associated and other soluble and non-soluble proteins.

In our study, we isolated abundant exosome particles from the VH of our cohort using differential ultracentrifugation and confirmed them as exosome-enriched EVs by their size, the number of particles, morphology, and exosome markers. A few studies have attempted to characterize EV or exosomes in VH; there has been inconsistency in the presence of traditional exosome markers. Ragusa et al. reported that CD63 and CD81 were present in their study from human VH exosomes from patients with melanoma. Zhao et al. reported that CD63 and CD81 were absent in their postmortem human VH. We confirmed both CD63 and CD81 were present in all VH exosomes [22,23]. Further, the previous studies on VH exosomes often focused on identifying a disease biomarker from ocular tumors [22,24]. However, very little is known about the role of exosomes in the development of ocular pathology and studies on exosome-mediated cell-to-cell communication in ocular pathologies are still in the early stages [21,38]. In the present study, we demonstrated that exosome proteins from VH with PVR were related to known PVR pathways, such as gliosis, cellular proliferation, inflammation, growth of connective tissues, and epithelial mesenchymal transition (EMT).

We further found that sets of significantly upregulated proteins in PVR, including SPP1, CLU, VCAN, COL2A1, and SEMA7A, were related to tissue remodeling. Because these key pathways are known to involve a complex set of cellular constituents, including Müller cells, astrocytes and microglia cells, retinal pigment epithelial cells (RPE), hyalocytes, inflammatory cells, and fibroblasts, it is speculated that exosomes are involved in PVR development through cell-to-cell communication [2,4,5,6,39]. This speculation is further supported by a recent study by Morris et al. that demonstrated that retinal pigment epithelium (RPE)-derived exosomal miRNA was transferred to microglia [40]. Their study demonstrated that exosomal cargo can be transferred to a different type of cell within the retina.

New therapeutic strategies blocking the cellular release or uptake of exosomes is an intriguing emerging concept because this approach may broadly target the coordinated complex cellular and pathologic processes. The lessons from several failed clinical trials targeting a selective PVR pathway, such as cellular proliferation or inflammation, suggest that a more comprehensive approach to target multi-pathways may be necessary to tackle PVR [11,12,13,14]. Our study shows that exosomes carry a unique compartment of proteins that are involved in many of the known PVR pathways, and the number of exosome particles that were increased in severe PVR further suggest that strategies that interfere with the genesis and/or secretion of exosomes and impair exosome-mediated cell-to-cell communication could potentially be a therapeutic approach for PVR. While much of the biology of exosome genesis and secretion in PVR needs to be determined before considering an exosome-targeted treatment strategy, there are several additional potential barriers to consider. Identifying specific exosome subtypes from different cellular origins or with different functional roles will be necessary to target exosomes that are involved in the pathologic process and to avoid interrupting the physiologic functions of the exosomes. In the present study, we identified frequent N-glycosylation of the exosomal proteins. Previously, several studies reported glycomic changes involving N-glycosylation associated with PVR or EMT [41,42]. Further studies to identify the role of N-glycosylation in exosomes and PVR are needed to identify targeted exosomes involved in PVR.

In our previous study, we demonstrated that elevated GFAP and vimentin in VH, hallmarks of retinal gliosis, were highly correlated with the clinical severity of PVR [15]. In the current study, we also identified GFAP and vimentin in our exosomes. However, we found that VH exosomes are not a major pool of GFAP or vimentin (data not shown). We speculate that GFAP and vimentin may be present in several pools, including the exosome and soluble pool of VH. Nevertheless, the previous literature reported 15 DE proteins between severe/mild PVR and controls that were associated with gliosis. PVR-unique proteins were highly associated with reactive gliosis in IPA analysis. While studies to explore the link between retinal gliosis and the PVR pathology are limited, we support that reactive retinal gliosis plays a role in PVR development where exosomes are involved.

The present study had limitations. The study included a relatively small number of patients, especially for RD with no PVR and mild PVR. Although we observed a distinctive difference between ERM/MH and severe PVR, proteomic changes in exosomes in RD with various stages of PVR may provide further insights to understand the development of PVR. Because billions of exosomes were present in our VH sample, proteomic analysis using liquid chromatography–tandem mass spectrometry (LC-MS/MS) is rather sensitive to determine protein changes. Nevertheless, a validation study in a larger cohort of patients will benefit to understand heterogenous molecular changes and variable clinical presentation during PVR development. As we discussed earlier, developing a new molecular change-based staging system for PVR may be necessary to understand PVR development and future clinical trials. Further, VH exosomes may not represent all exosomes that are intracellularly generated during the PVR development. Further studies on exosome genesis and secretion during the PVR development will be necessary.

In summary, we demonstrated a unique proteomic profiling from the exosomes released into the VH during PVR development. Abundant proteins in exosomes in PVR were highly related to tissue remodeling along with the known key PVR pathways, suggesting that exosomes may play a role in mediating complex cellular and diseases processes involved in PVR.

## Data Availability

All DE proteins, from the five pairwise disease stage comparisons, are listed in the supplementary file (genes diff.csv). Sample 1 and sample 2 refer to the pair of samples compared. IgFold stands for log fold change; test stat, *p* value, and q value are statistics from testing the strength of differentiation. q value (also called *fdr*) is the *p* value adjusted for multiple testing. A q value < 0.05 was used as the threshold for calling the difference significant. The remaining columns are protein annotations. The mass spectrometry data have been deposited to the ProteomeXchange Consortium via the PRIDE partner repository with the data set identifier PXD028539.

## Supplementary Materials

The following supporting information can be downloaded at: https://www.mdpi.com/article/10.3390/jcm11102716/s1, Table S1: A full list of proteins observed in each sample group; Table S2: A full list of differentially expressed proteins; Table S3: A full list of original expression values and clustering membership details in heat map.
