# Supplementary material for: Proteomic Insight into the Role of Exosomes in Proliferative Vitreoretinopathy Development
Source: J Clin Med. 2022 May 11;11(10):2716. doi: 10.3390/jcm11102716 (PMC9143131; doi:10.3390/jcm11102716)
Supplement: Supplementary file 1 [file jcm-11-02716-s001.zip › jcm-1508952-supplementary.pdf]

Supplementary Table S1

Proteins observed in each sample group ("+" indicates presence; "-" indicates absence)					
**Symbol**	**ERM**	**MH**	**RD no PVR**	**RD mild PVR**	**RD severe PVR**
**A1BG**	+	+	+	+	+
**A2M**	+	+	+	+	+
**ABCA4**	-	-	+	-	-
**ABCF1**	+	+	+	-	+
**ABHD14A**	-	-	-	-	+
**ABI3BP**	+	+	+	-	+
**ACADVL**	-	-	-	-	+
**ACAN**	+	+	+	-	-
**ACSM1**	+	-	-	-	-
**ACTA1**	-	-	-	-	+
**ACTB**	-	-	+	-	+
**ACTBL2**	-	+	-	-	-
**ACTG2**	-	-	-	-	+
**ADA2**	-	-	-	-	+
**ADCY6**	-	-	-	+	-
**ADGRB2**	-	-	-	-	+
**AFM**	+	+	+	+	+
**AGA**	-	+	+	-	+
**AGRN**	+	+	+	-	+
**AGT**	+	+	+	+	+
**AGXT2**	-	+	-	-	-
**AHSG**	+	+	+	+	+
**AKAP2**	+	-	-	-	-
**ALB**	+	+	+	+	+
**ALDOA**	-	-	+	-	+
**ALDOC**	+	+	+	-	+
**ALG13**	-	+	-	-	-
**AMBP**	+	+	+	-	+
**ANKRD18A**	-	+	-	-	-
**ANKRD26**	-	-	-	-	+
**ANKRD30BL**	-	-	-	-	+
**ANXA1**	-	-	-	-	+
**ANXA2**	-	-	-	+	-
**APCDD1**	-	-	+	-	+
**APCS**	-	+	-	-	+
**APLP1**	+	+	+	-	+
**APLP2**	+	+	+	-	+

Proteins observed in each sample group<br>("+" indicates presence; "-" indicates absence)					
Symbol	ERM	MH	RD no PVR	RD mild PVR	RD severe PVR
APOA1	+	+	+	+	+
APOA2	+	+	+	+	+
APOA4	+	+	+	+	+
APOB	-	+	-	-	+
APOC1	-	+	-	-	+
APOC2	-	-	-	-	+
APOC3	+	+	+	-	+
APOD	+	+	+	-	+
APOE	+	+	+	+	+
APOF	+	+	-	-	+
APOH	+	+	+	-	+
APOL1	-	+	-	-	-
APOM	+	-	-	-	+
APP	-	+	+	-	+
ARHGAP5	-	-	+	-	-
ASAH1	+	+	+	-	-
ASAP2	-	-	-	-	+
ATP1A1	-	-	+	-	-
ATP6AP1	+	+	+	-	+
ATRN	+	+	+	-	+
ATXN10	-	-	-	-	+
AZGP1	+	+	+	+	+
B2M	+	+	+	-	+
B4GAT1	+	+	+	-	+
BAX	-	-	-	+	+
BCAN	+	+	-	-	-
BCAP29	-	-	+	-	-
BCHE	-	+	-	-	+
BNIP5	-	-	-	-	+
BTBD16	+	+	-	-	-
BTD	+	+	+	-	+
BUB1	-	-	-	-	+
C1QA	-	-	-	-	+
C1QB	+	+	+	-	+
C1QC	+	+	+	-	+
C1QTNF3	-	-	-	-	+
C1R	+	+	+	-	+
C1RL	+	-	-	-	-
C1S	+	+	+	-	+

Proteins observed in each sample group ("+" indicates presence; "-" indicates absence)					
Symbol	ERM	MH	RD no PVR	RD mild PVR	RD severe PVR
C2	+	+	+	-	+
C3	+	+	+	+	+
C4A	+	+	+	+	+
C4B	+	+	+	-	+
C4BPA	-	+	-	-	+
C4BPB	-	+	-	-	-
C5	+	+	+	-	+
C6	+	+	+	-	+
C7	+	+	+	-	+
C8A	+	+	+	-	+
C8B	+	+	+	-	+
C8G	+	+	+	-	+
C8orf37	-	-	-	+	+
C9	+	+	+	+	+
CA10	-	+	-	-	-
CA11	-	-	-	-	+
CACNA1D	-	+	-	-	-
CADM1	+	+	+	-	+
CADM2	+	+	-	-	-
CALM3	+	+	+	-	+
CCDC88C	+	-	-	-	-
CD14	+	+	+	-	+
CD163	+	-	-	-	+
CD22	-	-	-	-	+
CD44	-	+	-	-	+
CD59	+	+	+	-	+
CD5L	-	-	-	-	+
CD63	-	-	+	-	-
CD81	-	-	-	-	+
CDH1	-	-	-	-	+
CDH2	+	+	+	-	+
CDHR1	+	+	+	-	+
CDK5RAP2	-	-	-	-	+
CELSR1	-	-	+	-	-
CENPVL3	+	-	-	-	-
CETP	-	+	-	-	-
CFB	+	+	+	+	+
CFD	+	+	+	-	+
CFH	+	+	+	-	+

Proteins observed in each sample group<br>("+" indicates presence; "-" indicates absence)					
Symbol	ERM	MH	RD no PVR	RD mild PVR	RD severe PVR
CFHR1	+	+	+	-	+
CFHR2	+	+	+	-	+
CFI	+	+	+	-	+
CFL1	-	-	+	-	-
CHGA	+	+	+	-	+
CHGB	-	-	-	-	+
CHI3L1	+	+	+	+	+
CHL1	+	+	+	-	+
CHRDL1	+	+	+	-	+
CKB	-	-	+	-	+
CLEC3B	+	+	+	-	+
CLN5	-	+	+	-	-
CLSTN1	+	+	+	-	+
CLSTN2	-	+	+	-	-
CLSTN3	-	+	+	-	+
CLU	+	+	+	+	+
CLUL1	-	+	+	-	+
CMTM1	-	-	+	-	-
CNDP1	+	+	+	-	+
CNTN1	+	+	+	-	+
CNTN2	-	+	-	-	-
CNTN4	-	+	-	-	-
CNTN6	-	-	-	-	+
COL11A1	+	-	+	-	-
COL18A1	+	+	+	-	+
COL1A1	-	+	-	-	-
COL1A2	+	+	+	-	-
COL22A1	-	-	+	-	-
COL2A1	+	+	+	-	+
COL6A1	+	+	+	-	+
COL9A1	+	+	+	-	+
COL9A2	+	+	+	-	+
COLEC12	-	-	-	-	+
COTL1	-	-	-	-	+
CP	+	+	+	+	+
CPAMD8	+	+	+	-	+
CPB2	-	+	-	-	+
CPE	+	+	+	-	+
CPN1	+	+	-	-	+

Proteins observed in each sample group ("+" indicates presence; "-" indicates absence)					
**Symbol**	**ERM**	**MH**	**RD no PVR**	**RD mild PVR**	**RD severe PVR**
**CPN2**	+	+	-	-	+
**CPQ**	+	+	+	-	+
**CPT1B**	-	-	-	-	+
**CPVL**	-	+	+	-	+
**CRB1**	-	-	-	+	+
**CRISP3**	-	-	-	-	+
**CRP**	+	+	+	-	+
**CRTAC1**	+	+	+	-	+
**CRYAA**	+	+	-	-	+
**CRYAB**	-	-	-	-	+
**CRYBA1**	-	+	-	-	-
**CRYBB1**	-	+	-	-	-
**CRYBB2**	+	+	+	-	+
**CRYGS**	-	+	-	-	-
**CSF1**	-	-	+	-	-
**CSF1R**	-	-	+	-	+
**CSPG5**	-	-	+	-	-
**CST3**	+	+	+	-	+
**CTBS**	-	+	-	-	-
**CTSA**	+	+	+	-	-
**CTSB**	+	+	+	-	+
**CTSD**	+	+	+	+	+
**CTSF**	-	+	-	-	+
**CTSL**	-	+	+	-	-
**CTSZ**	+	+	+	-	+
**CUTA**	+	+	+	-	+
**CWC27**	-	-	-	+	-
**CXCL16**	-	-	+	-	+
**CYCS**	-	+	-	-	+
**DAG1**	+	+	+	-	+
**DBF4**	-	-	+	-	-
**DCD**	-	-	-	+	-
**DCN**	-	-	+	-	-
**DDX1**	-	-	-	-	+
**DDX51**	-	-	-	-	+
**DEFA1**	-	-	-	-	+
**DIAPH1**	+	-	-	-	-
**DIPK1C**	-	+	+	-	-
**DKK3**	+	+	+	-	+

Proteins observed in each sample group ("+" indicates presence; "-" indicates absence)					
Symbol	ERM	MH	RD no PVR	RD mild PVR	RD severe PVR
DNAH10	-	+	-	-	-
DNAH3	-	-	-	-	+
DNASE2	+	+	+	-	-
DNER	-	+	+	-	+
DPP7	+	+	+	-	+
DSG1	+	+	+	+	+
DSG2	-	-	-	-	+
DSP	+	-	-	+	+
DYNLRB2	-	+	-	-	-
ECM1	+	+	+	-	+
EEF1A2	-	-	-	-	+
EFEMP1	+	+	+	-	+
EGFLAM	-	-	+	-	-
EMILIN1	-	+	-	-	-
ENO1	-	-	-	-	+
ENO2	-	+	+	-	+
ENO3	-	+	+	-	-
ENPP2	+	+	+	-	+
ENTPD2	-	+	-	-	-
EPDR1	+	+	+	-	+
EPHB1	-	-	-	-	+
ERMARD	-	-	-	-	+
ERN1	-	+	-	-	+
ERVK-7	-	-	-	-	+
EXO1	-	-	-	-	+
EZR	-	-	-	-	+
F10	-	-	-	-	+
F12	+	+	+	-	+
F13A1	-	-	-	-	+
F13B	+	+	-	-	+
F2	+	+	+	-	+
F5	+	+	+	-	+
F9	+	+	-	-	+
FABP5	+	+	+	-	-
FAM3C	+	+	+	-	+
FBLN1	+	+	+	-	+
FBN1	+	+	+	-	+
FBP1	-	-	-	-	+
FCGBP	+	+	+	+	+

Proteins observed in each sample group ("+" indicates presence; "-" indicates absence)					
**Symbol**	**ERM**	**MH**	**RD no PVR**	**RD mild PVR**	**RD severe PVR**
**FER1L5**	-	-	-	-	+
**FETUB**	+	+	+	-	+
**FGA**	+	+	+	+	+
**FGB**	+	+	+	-	+
**FGG**	+	+	+	+	+
**FLG2**	-	-	-	+	+
**FN1**	+	+	+	-	+
**FRMPD1**	-	-	+	-	-
**FRZB**	+	+	+	-	+
**FSTL1**	+	+	+	-	+
**FSTL4**	-	+	+	-	-
**FSTL5**	+	+	+	-	+
**FTL**	-	-	-	-	+
**FUCA1**	-	+	-	-	+
**FUCA2**	-	+	+	-	-
**GAA**	+	+	+	-	+
**GAPDH**	+	+	+	+	+
**GAS6**	-	+	-	-	-
**GBA**	-	+	-	-	+
**GC**	+	+	+	+	+
**GDI1**	-	-	-	-	+
**GFAP**	-	-	-	-	+
**GGH**	-	+	-	-	-
**GIPC2**	-	-	-	-	+
**GKN1**	-	-	-	-	+
**GM2A**	-	-	+	-	-
**GNAS**	-	-	+	-	-
**GNAT1**	-	-	+	-	-
**GNB1**	-	-	+	-	+
**GNGT1**	-	-	-	-	+
**GNPTG**	-	-	-	-	+
**GNS**	+	+	+	-	+
**GOLIM4**	-	+	-	-	-
**GOLM1**	-	+	+	-	+
**GOT2**	+	-	-	-	-
**GPI**	-	-	+	-	+
**GPLD1**	-	-	-	-	+
**GPM6A**	-	-	+	-	+
**GPX3**	+	+	+	+	+

Proteins observed in each sample group ("+" indicates presence; "-" indicates absence)					
Symbol	ERM	MH	RD no PVR	RD mild PVR	RD severe PVR
GRIA4	+	+	-	-	+
GSN	+	+	+	+	+
GSTP1	-	-	-	-	+
GTF3C5	-	-	+	-	+
H2AC20	-	-	+	-	-
H4C1	-	-	+	-	+
HABP2	+	+	+	-	+
HARS2	-	+	-	-	-
HBA1	+	+	+	-	+
HBB	+	+	+	-	+
HBD	-	+	-	-	-
HEATR1	-	-	-	-	+
HEBP2	-	-	-	-	+
HEXA	-	-	+	-	+
HEXB	-	-	+	-	-
HGFAC	+	+	-	-	+
HOMER2	-	-	-	-	+
HP	+	+	+	+	+
HPR	+	+	-	-	+
HPX	+	+	+	+	+
HRG	+	+	+	+	+
HSP90AA1	-	-	+	-	+
HSP90B1	-	-	-	-	+
HSPA13	-	-	+	-	+
HSPA1B	-	-	+	-	-
HSPA4	-	+	-	+	-
HSPA5	+	+	+	-	-
HSPA7	+	-	-	-	-
HSPA8	-	-	+	-	-
HSPG2	+	+	+	-	+
HTRA1	-	-	+	-	+
HYDIN	-	+	-	-	-
HYI	-	-	-	-	+
HYOU1	-	+	-	-	-
ICOSLG	-	-	-	-	+
IDH1	-	-	-	-	+
IDS	-	+	+	-	-
IFT46	-	+	-	-	-
IGF2	+	+	+	-	+

Proteins observed in each sample group ("+" indicates presence; "-" indicates absence)					
Symbol	ERM	MH	RD no PVR	RD mild PVR	RD severe PVR
IGFALS	+	+	+	-	+
IGFBP2	+	+	+	-	+
IGFBP3	+	-	+	-	+
IGFBP4	-	+	+	-	-
IGFBP5	-	+	+	-	+
IGFBP6	+	+	+	-	+
IGFBP7	+	+	+	-	+
IGFN1	-	+	-	-	-
IGHA1	+	+	+	+	+
IGHA2	+	+	+	+	+
IGHD	+	+	+	-	+
IGHG1	+	+	+	+	+
IGHG2	+	+	-	+	+
IGHG3	+	+	+	+	+
IGHG4	+	+	+	+	+
IGHM	-	+	-	-	+
IGHV1-2	-	-	+	-	-
IGHV1-3	-	+	+	-	-
IGHV1-69	+	+	+	+	+
IGHV1OR15-1	-	-	-	+	+
IGHV2-70D	+	+	-	-	+
IGHV3-13	+	+	+	-	+
IGHV3-15	-	+	-	-	+
IGHV3-30	+	+	+	+	+
IGHV3-35	+	+	+	-	+
IGHV3-38	-	-	-	-	+
IGHV3-43	+	-	-	-	+
IGHV3-43D	+	+	+	-	+
IGHV3-48	+	+	+	-	+
IGHV3-49	+	+	+	-	+
IGHV3-64D	+	+	-	-	+
IGHV3-7	+	+	+	+	+
IGHV3-72	+	+	+	-	+
IGHV3-73	-	+	-	-	-
IGHV3-74	-	+	-	+	+
IGHV4-28	+	-	-	-	-
IGHV4-34	+	+	+	+	+
IGHV4-4	-	-	-	-	+
IGHV5-10-1	-	+	+	-	+

Proteins observed in each sample group<br>("+" indicates presence; "-" indicates absence)					
**Symbol**	**ERM**	**MH**	**RD no PVR**	**RD mild PVR**	**RD severe PVR**
**IGHV5-51**	+	+	-	-	+
**IGHV6-1**	+	+	+	-	+
**IGKC**	+	+	+	+	+
**IGKV1-16**	-	+	-	-	+
**IGKV1-17**	+	+	-	-	+
**IGKV1-27**	-	-	-	-	+
**IGKV1-5**	+	+	+	-	+
**IGKV1-6**	-	-	-	-	+
**IGKV1D-13**	+	+	-	-	+
**IGKV1D-33**	+	+	+	-	+
**IGKV2-30**	-	+	-	-	-
**IGKV2-40**	+	+	+	+	+
**IGKV2D-24**	+	+	+	-	+
**IGKV3-15**	+	+	+	-	+
**IGKV3-20**	-	+	+	+	+
**IGKV3-7**	-	+	-	-	+
**IGKV3D-11**	+	+	+	+	+
**IGKV3D-15**	-	+	-	-	+
**IGKV3D-20**	+	+	-	-	+
**IGKV4-1**	+	+	-	+	+
**IGKV6D-41**	+	+	-	-	-
**IGLC2**	+	+	+	+	+
**IGLC7**	-	-	-	+	-
**IGLL1**	-	-	-	+	+
**IGLL5**	+	+	+	-	+
**IGLV1-40**	-	-	-	-	+
**IGLV1-44**	-	+	-	-	+
**IGLV1-47**	+	+	+	+	+
**IGLV1-51**	+	+	-	-	+
**IGLV2-14**	-	+	-	-	+
**IGLV2-18**	+	+	-	-	+
**IGLV3-1**	-	+	-	-	-
**IGLV3-10**	+	+	+	-	+
**IGLV3-19**	-	-	+	-	+
**IGLV3-21**	+	+	+	+	+
**IGLV4-69**	-	+	-	-	-
**IGLV5-39**	-	-	-	-	+
**IGLV6-57**	-	+	-	-	+
**IGLV7-46**	+	-	+	-	-

Proteins observed in each sample group<br>("+" indicates presence; "-" indicates absence)					
Symbol	ERM	MH	RD no PVR	RD mild PVR	RD severe PVR
IGLV8-61	+	+	-	-	+
IL6ST	-	-	+	-	-
IMMT	-	-	+	-	-
IMPG1	+	-	+	+	+
IMPG2	+	+	+	+	+
INA	-	-	-	-	+
INPP4B	-	-	+	-	-
IRAK4	-	-	-	-	+
ITGA8	-	+	-	-	+
ITIH1	+	+	+	+	+
ITIH2	+	+	+	+	+
ITIH3	+	+	-	-	-
ITIH4	+	+	+	-	+
ITIH5	+	+	+	-	-
ITM2B	-	+	-	-	-
JCHAIN	-	+	-	-	+
JUP	-	-	-	+	+
KARS1	-	-	+	-	+
KEAP1	-	-	-	-	+
KIF15	-	-	+	-	-
KIF7	-	+	-	-	-
KLKB1	+	+	-	-	+
KNG1	+	+	+	-	+
KPRP	-	-	-	+	+
KRT1	+	+	+	+	+
KRT10	+	+	+	+	+
KRT12	-	-	+	-	+
KRT13	-	-	-	-	+
KRT14	+	+	+	+	+
KRT15	-	+	+	-	+
KRT16	-	-	-	+	+
KRT17	-	-	-	+	+
KRT2	+	+	+	+	+
KRT25	-	-	-	+	+
KRT27	-	-	-	+	+
KRT28	-	-	-	+	-
KRT31	-	-	-	-	+
KRT4	-	-	-	-	+
KRT5	+	+	-	+	+

Proteins observed in each sample group ("+" indicates presence; "-" indicates absence)					
**Symbol**	**ERM**	**MH**	**RD no PVR**	**RD mild PVR**	**RD severe PVR**
**KRT6A**	-	-	-	+	+
**KRT6B**	-	-	-	+	+
**KRT7**	-	-	-	+	-
**KRT71**	-	-	-	+	+
**KRT73**	-	-	-	+	+
**KRT74**	+	+	-	-	-
**KRT77**	-	-	-	+	+
**KRT78**	-	-	-	+	+
**KRT79**	-	-	-	-	+
**KRT81**	-	+	-	-	+
**KRT9**	+	+	+	+	+
**LAMP2**	-	+	+	-	+
**LBP**	+	+	-	-	+
**LCA5L**	-	-	-	-	+
**LCN1**	+	+	+	+	+
**LCN2**	+	+	+	-	+
**LCP1**	-	-	+	-	+
**LDHA**	-	-	-	-	+
**LDHC**	-	-	+	-	-
**LENG8**	-	+	-	-	-
**LGALS3BP**	+	+	+	+	+
**LIN52**	+	-	-	-	-
**LMAN2**	-	+	+	-	-
**LPA**	-	+	-	-	+
**LRBA**	+	-	-	-	-
**LRG1**	+	+	+	+	+
**LRIT1**	+	+	+	-	-
**LRP1**	-	+	+	-	+
**LRP2**	+	+	+	-	+
**LSAMP**	-	+	+	-	+
**LTBP2**	+	+	+	-	+
**LTF**	+	-	-	-	-
**LUM**	+	+	+	-	+
**LYVE1**	-	-	-	-	+
**LYZ**	+	+	+	+	+
**MACF1**	-	-	+	-	-
**MAN1A1**	-	+	+	-	-
**MAN1C1**	-	-	-	-	+
**MAN2A1**	-	-	+	-	+

Proteins observed in each sample group ("+" indicates presence; "-" indicates absence)					
Symbol	ERM	MH	RD no PVR	RD mild PVR	RD severe PVR
MAN2A2	-	-	+	-	+
MANBA	-	+	+	-	+
MASP2	-	-	-	-	+
MDH1	-	-	+	-	-
MDN1	-	-	-	-	+
MEGF8	-	+	-	-	+
MET	-	-	+	-	+
METTL25	-	+	-	-	-
MFAP4	-	+	+	-	+
MFGE8	-	+	+	-	-
MGAM	-	-	-	+	-
MICAL2	-	-	-	+	-
MIF	-	-	-	-	+
MINPP1	-	+	+	-	+
MMP2	+	+	+	-	+
MRPS31	-	+	-	-	-
MSH2	-	-	+	-	-
MSLN	-	-	-	-	+
MSN	-	-	+	-	+
MST1	+	+	+	-	-
MST1L	-	+	-	-	-
MUC19	-	-	+	-	-
MYOC	+	+	+	-	+
NAGA	+	+	+	-	-
NAGLU	-	-	+	-	+
NBAS	-	-	-	-	+
NCAM1	+	+	+	-	+
NCAN	+	+	+	-	-
NDST4	-	-	-	-	+
NEGR1	-	+	+	-	+
NELL2	-	+	+	-	+
NEO1	+	+	+	-	+
NEU1	-	+	-	-	-
NFASC	+	+	-	-	+
NGFR	+	-	+	-	-
NIPBL	-	-	-	-	+
NOL4	-	-	-	-	+
NOTCH2NLB	-	-	+	-	-
NPC2	+	+	+	-	+

Proteins observed in each sample group<br>("+" indicates presence; "-" indicates absence)					
Symbol	ERM	MH	RD no PVR	RD mild PVR	RD severe PVR
NPTX1	-	+	-	-	-
NPY6R	-	-	-	+	+
NRCAM	+	+	+	-	+
NRXN3	+	+	+	-	+
NT5C3B	-	+	+	+	+
NTM	-	+	+	-	+
NUCB1	+	+	+	-	+
NUMA1	-	-	-	+	-
OAF	+	+	+	-	+
OCRL	-	-	-	-	+
OLFM1	-	+	+	-	+
OLFM2	-	+	+	-	+
OMG	-	-	+	-	-
OPTC	+	+	+	+	+
ORM1	+	+	+	+	+
ORM2	+	+	+	+	+
P4HA3	-	+	-	-	-
PAM	-	+	-	-	-
PAPLN	-	-	-	-	+
PAPPA2	+	+	+	-	+
PCOLCE	+	+	-	-	-
PCSK1N	+	+	+	-	+
PCSK2	-	-	+	-	-
PDCD6IP	-	-	+	-	-
PDE2A	-	-	-	-	+
PDE6A	-	-	+	-	-
PDE6B	-	-	+	-	-
PDE8A	-	+	-	-	-
PDIA3	-	-	+	-	+
PEBP1	+	+	+	-	+
PEBP4	+	+	-	-	+
PGAM4	+	-	-	-	+
PGK1	+	+	+	-	+
PGK2	-	-	+	-	-
PGLYRP2	+	+	+	-	+
PI16	-	-	+	-	-
PICK1	-	-	-	-	+
PIK3IP1	+	+	+	-	+
PIP	-	-	-	+	-

Proteins observed in each sample group ("+" indicates presence; "-" indicates absence)					
Symbol	ERM	MH	RD no PVR	RD mild PVR	RD severe PVR
PKM	+	+	+	-	+
PKP1	-	-	-	-	+
PLA1A	-	-	-	-	+
PLD3	+	+	+	-	-
PLG	+	+	+	-	+
PLTP	+	+	+	-	+
PLXDC2	-	+	-	-	-
PLXNB2	+	+	+	-	+
PMS2CL	-	+	-	-	-
POLQ	-	-	-	-	+
POLR2A	-	-	-	-	+
PON1	+	+	+	-	+
POTEF	+	+	-	+	+
PPT1	-	-	+	-	-
PRDX1	-	-	+	-	+
PRDX2	-	-	+	-	-
PRKCSH	-	+	+	-	-
PRNP	+	+	+	-	+
PROM1	-	-	+	-	+
PROS1	+	+	+	-	+
PRPF8	-	-	+	-	-
PRSS1	-	-	-	+	+
PRSS3	-	-	-	+	-
PRSS42P	-	-	-	-	+
PSAP	+	+	+	-	+
PSD	-	-	-	-	+
PTGDS	+	+	+	+	+
PTPN4	-	-	-	-	+
PTPRF	-	+	+	-	-
PTPRG	-	-	+	-	-
PTPRQ	-	-	-	-	+
PTPRZ1	+	+	+	-	+
PTX3	-	-	+	-	+
PXDN	-	-	-	-	+
PYGM	-	+	+	-	-
PZP	+	+	-	-	+
QPCT	+	+	-	-	+
QSOX1	+	+	+	-	-
QSOX2	-	-	-	-	+

Proteins observed in each sample group ("+" indicates presence; "-" indicates absence)					
Symbol	ERM	MH	RD no PVR	RD mild PVR	RD severe PVR
RAB11FIP1	+	+	-	-	-
RAB3IL1	-	+	-	-	-
RARRES2	+	+	+	-	+
RBM26	-	-	-	-	+
RBP1	-	-	+	-	+
RBP3	+	+	+	+	+
RBP4	+	+	+	-	+
RDX	-	-	+	-	-
RELN	-	+	-	-	-
RHO	-	-	+	-	-
RHPN2P1	-	-	-	-	+
RIC8B	-	-	+	-	+
RLBP1	-	-	+	-	+
RMND5A	-	-	-	-	+
RNASE1	+	+	+	-	+
RNASET2	-	+	+	-	+
RS1	+	+	+	-	+
RTBDN	+	+	+	-	+
S100A8	-	-	-	+	-
S100A9	-	-	-	+	+
SAA2	-	+	-	-	-
SAA4	-	+	+	-	+
SAAL1	-	-	-	-	+
SAG	-	-	+	-	-
SAMD15	-	+	-	-	-
SCFD1	-	-	-	-	+
SCG2	-	+	+	-	-
SCG3	+	+	+	-	-
SCG5	+	+	-	-	-
SDCBP	-	-	+	-	+
SEC22B	-	-	-	-	+
SELENBP1	-	-	-	-	+
SELENOP	+	+	+	-	+
SEMA3F	-	+	+	-	+
SEMA4B	-	-	+	-	+
SEMA7A	+	+	+	-	+
SEPTIN4	-	-	-	-	+
SERPINA1	+	+	+	+	+
SERPINA10	+	+	-	-	+

Proteins observed in each sample group ("+" indicates presence; "-" indicates absence)					
**Symbol**	**ERM**	**MH**	**RD no PVR**	**RD mild PVR**	**RD severe PVR**
**SERPINA3**	+	+	+	+	+
**SERPINA4**	+	+	+	-	+
**SERPINA5**	+	+	+	-	+
**SERPINA6**	+	+	+	-	+
**SERPINA7**	+	+	+	-	+
**SERPINB12**	-	-	-	+	+
**SERPINC1**	+	+	+	+	+
**SERPIND1**	+	+	+	-	+
**SERPINF1**	+	+	+	+	+
**SERPINF2**	+	+	+	-	+
**SERPING1**	+	+	+	+	+
**SERPINI1**	+	+	+	-	+
**SEZ6**	-	+	+	-	-
**SEZ6L**	-	+	+	-	-
**SFRP4**	+	+	-	-	-
**SHBG**	+	+	-	-	+
**SIAE**	+	+	+	-	+
**SLC20A2**	-	-	-	+	-
**SLC2A1**	-	-	-	-	+
**SLC35C2**	-	-	-	-	+
**SLC39A12**	-	-	-	-	+
**SLC3A2**	-	-	+	-	+
**SLC8A2**	-	+	-	-	-
**SMARCC1**	-	+	-	-	-
**SMPDL3A**	-	-	+	-	+
**SOD1**	+	+	+	-	+
**SOD3**	+	+	+	-	+
**SORCS1**	+	-	-	-	-
**SOX10**	-	-	-	-	+
**SPARC**	+	+	+	-	-
**SPARCL1**	+	+	+	-	+
**SPEN**	-	+	-	-	-
**SPIB**	-	-	-	-	+
**SPOCK1**	+	+	+	-	+
**SPOCK2**	-	+	-	-	-
**SPOCK3**	-	+	-	-	-
**SPON1**	+	+	+	-	+
**SPP1**	+	+	+	-	+
**SPTBN5**	-	-	-	-	+

Proteins observed in each sample group ("+" indicates presence; "-" indicates absence)					
Symbol	ERM	MH	RD no PVR	RD mild PVR	RD severe PVR
SRBD1	-	+	-	-	-
SRPRA	-	-	-	-	+
TADA2B	-	-	-	+	+
TAF4	-	-	-	-	+
TALDO1	-	-	+	-	-
TCP11L1	-	-	-	-	+
TENM3	+	-	-	-	-
TF	+	+	+	+	+
TFAP2B	-	-	+	-	-
TGFBI	+	+	+	-	+
TGIF2	-	-	-	-	+
TGM1	-	-	-	+	-
TGM7	-	-	-	-	+
TIMP1	+	+	+	-	+
TIMP2	-	+	-	-	-
TLN1	-	-	-	-	+
TMBIM6	+	+	-	-	+
TMEM198	-	-	-	-	+
TNC	+	+	+	-	-
TNR	-	+	+	-	+
TNRC6C	-	-	+	-	-
TPI1	+	+	+	-	+
TPP1	+	+	+	-	+
TPST1	-	-	-	+	-
TRANK1	-	-	-	-	+
TRMT1L	-	+	-	-	-
TRPC1	-	-	-	-	+
TRPM6	-	-	-	-	+
TTBK1	-	+	-	-	-
TTC27	-	+	-	-	-
TTC7A	-	-	-	-	+
TTR	+	+	+	+	+
TUBA3D	-	-	+	-	+
TUBB2A	-	-	-	-	+
TWSG1	-	-	+	-	-
TXN	-	-	-	+	-
TYMP	-	-	-	-	+
TYRP1	-	-	+	-	-
UBA52	+	-	+	+	+

Proteins observed in each sample group ("+" indicates presence; "-" indicates absence)					
Symbol	ERM	MH	RD no PVR	RD mild PVR	RD severe PVR
UGT2A2	-	-	-	-	+
UGT8	-	+	-	-	-
UNC45A	-	-	-	-	+
VASN	+	+	+	-	+
VCAN	+	+	+	-	+
VGF	-	-	+	-	-
VIM	+	-	+	-	+
VNN1	-	-	-	-	+
VPS54	-	-	-	-	+
VTN	+	+	+	+	+
WARS1	-	+	+	-	-
WFIKKN2	-	+	-	-	-
WIF1	+	+	+	-	-
XYLT1	-	-	+	-	-
YWHAE	-	-	-	-	+
YWHAZ	-	-	-	-	+
ZBED4	-	+	-	-	-
ZBTB40	-	-	-	-	+
ZBTB8B	-	-	+	-	-
ZG16B	-	-	-	-	+
ZNF266	-	-	-	+	-
ZNF33B	+	+	-	-	+
ZNF407	-	-	+	-	-
ZSWIM9	+	+	-	-	-

Supplementary Table S2

Differentially Expressed proteins			
Comparison	SYMBOL	logFold	p-value
RD vs. (ERM or MH)	C4A	-3.22	0.00038
RD vs. (ERM or MH)	KRT6A	-1.92	0.0028
RD vs. (ERM or MH)	KRT6B	-1.75	0.0021
RD vs. (ERM or MH)	KRT16	-1.57	0.0074
RD vs. (ERM or MH)	KRT77	-1.54	0.0021
RD vs. (ERM or MH)	IGHG4	-1.48	0.002
RD vs. (ERM or MH)	IGKC	-1.22	0.00026
RD vs. (ERM or MH)	KRT71	-1.18	0.0076

Differentially Expressed proteins			
**Comparison**	**SYMBOL**	**logFold**	**p-value**
**RD vs. (ERM or MH)**	**IMPG1**	-1.11	0.027
**RD vs. (ERM or MH)**	**CP**	-0.83	0.019
**RD vs. (ERM or MH)**	**ALB**	0.66	0.017
**RD vs. (ERM or MH)**	**SERPINA7**	0.75	0.016
**RD vs. (ERM or MH)**	**FSTL1**	0.79	0.018
**RD vs. (ERM or MH)**	**CD59**	0.79	0.026
**RD vs. (ERM or MH)**	**C1QB**	0.8	0.024
**RD vs. (ERM or MH)**	**CPB2**	0.8	0.026
**RD vs. (ERM or MH)**	**AFM**	0.8	0.028
**RD vs. (ERM or MH)**	**IGKV1D-33**	0.81	0.011
**RD vs. (ERM or MH)**	**IGFBP5**	0.81	0.023
**RD vs. (ERM or MH)**	**GSTP1**	0.81	0.027
**RD vs. (ERM or MH)**	**LDHA**	0.81	0.027
**RD vs. (ERM or MH)**	**AGA**	0.82	0.022
**RD vs. (ERM or MH)**	**DNER**	0.82	0.022
**RD vs. (ERM or MH)**	**OLFM1**	0.82	0.022
**RD vs. (ERM or MH)**	**SEMA3F**	0.82	0.022
**RD vs. (ERM or MH)**	**PTPN4**	0.84	0.02
**RD vs. (ERM or MH)**	**YWHAE**	0.84	0.02
**RD vs. (ERM or MH)**	**DSG2**	0.84	0.021
**RD vs. (ERM or MH)**	**IGHV3-38**	0.84	0.021
**RD vs. (ERM or MH)**	**ACTG2**	0.84	0.027
**RD vs. (ERM or MH)**	**CFHR1**	0.85	0.016
**RD vs. (ERM or MH)**	**IGLV3-10**	0.85	0.02
**RD vs. (ERM or MH)**	**IGKV3-7**	0.85	0.025
**RD vs. (ERM or MH)**	**RTBDN**	0.86	0.016
**RD vs. (ERM or MH)**	**IDS**	0.87	0.014
**RD vs. (ERM or MH)**	**MAN1A1**	0.87	0.014
**RD vs. (ERM or MH)**	**HSP90B1**	0.87	0.015
**RD vs. (ERM or MH)**	**TUBB2A**	0.87	0.015
**RD vs. (ERM or MH)**	**CRYAB**	0.87	0.018
**RD vs. (ERM or MH)**	**F13A1**	0.87	0.018
**RD vs. (ERM or MH)**	**LRG1**	0.87	0.019
**RD vs. (ERM or MH)**	**PLD3**	0.87	0.027
**RD vs. (ERM or MH)**	**F12**	0.88	0.022
**RD vs. (ERM or MH)**	**ACADVL**	0.88	0.024
**RD vs. (ERM or MH)**	**KRT31**	0.88	0.024
**RD vs. (ERM or MH)**	**IGHV3-49**	0.89	0.0082
**RD vs. (ERM or MH)**	**APOC2**	0.89	0.014

Differentially Expressed proteins			
**Comparison**	**SYMBOL**	**logFold**	**p-value**
**RD vs. (ERM or MH)**	**CD5L**	0.89	0.014
**RD vs. (ERM or MH)**	**F10**	0.89	0.014
**RD vs. (ERM or MH)**	**LYVE1**	0.89	0.014
**RD vs. (ERM or MH)**	**GFAP**	0.89	0.016
**RD vs. (ERM or MH)**	**CD163**	0.89	0.02
**RD vs. (ERM or MH)**	**APOM**	0.9	0.0095
**RD vs. (ERM or MH)**	**PGAM4**	0.9	0.0095
**RD vs. (ERM or MH)**	**PGK1**	0.9	0.012
**RD vs. (ERM or MH)**	**CFD**	0.91	0.0082
**RD vs. (ERM or MH)**	**GNGT1**	0.91	0.013
**RD vs. (ERM or MH)**	**CRP**	0.91	0.016
**RD vs. (ERM or MH)**	**LCN2**	0.91	0.02
**RD vs. (ERM or MH)**	**IGFBP6**	0.91	0.026
**RD vs. (ERM or MH)**	**IGHV2-70D**	0.92	0.0095
**RD vs. (ERM or MH)**	**APOC1**	0.93	0.013
**RD vs. (ERM or MH)**	**SCG2**	0.93	0.018
**RD vs. (ERM or MH)**	**JCHAIN**	0.94	0.011
**RD vs. (ERM or MH)**	**PROS1**	0.94	0.012
**RD vs. (ERM or MH)**	**SOD1**	0.94	0.014
**RD vs. (ERM or MH)**	**B2M**	0.95	0.027
**RD vs. (ERM or MH)**	**C1R**	0.96	0.0053
**RD vs. (ERM or MH)**	**SCG3**	0.96	0.015
**RD vs. (ERM or MH)**	**ACAN**	0.96	0.019
**RD vs. (ERM or MH)**	**GPX3**	0.97	0.012
**RD vs. (ERM or MH)**	**F13B**	0.97	0.012
**RD vs. (ERM or MH)**	**RS1**	0.98	0.0067
**RD vs. (ERM or MH)**	**CHRDL1**	0.98	0.016
**RD vs. (ERM or MH)**	**NUCB1**	0.99	0.0098
**RD vs. (ERM or MH)**	**C9**	0.99	0.011
**RD vs. (ERM or MH)**	**APOH**	0.99	0.015
**RD vs. (ERM or MH)**	**IGHV3-72**	0.99	0.027
**RD vs. (ERM or MH)**	**HGFAC**	1	0.0054
**RD vs. (ERM or MH)**	**IGKV3D-15**	1	0.0069
**RD vs. (ERM or MH)**	**FGB**	1	0.018
**RD vs. (ERM or MH)**	**IGKV3D-11**	1.01	0.0045
**RD vs. (ERM or MH)**	**MST1**	1.01	0.0069
**RD vs. (ERM or MH)**	**CNTN1**	1.01	0.011
**RD vs. (ERM or MH)**	**IGFBP4**	1.01	0.013
**RD vs. (ERM or MH)**	**PTPRF**	1.01	0.013

Differentially Expressed proteins			
**Comparison**	**SYMBOL**	**logFold**	**p-value**
**RD vs. (ERM or MH)**	**CLUL1**	1.01	0.017
**RD vs. (ERM or MH)**	**IGKV1-17**	1.03	0.0031
**RD vs. (ERM or MH)**	**IGLV2-18**	1.03	0.0031
**RD vs. (ERM or MH)**	**IGHV1-3**	1.03	0.005
**RD vs. (ERM or MH)**	**PON1**	1.04	0.0057
**RD vs. (ERM or MH)**	**CRTAC1**	1.05	0.0044
**RD vs. (ERM or MH)**	**CTSD**	1.05	0.012
**RD vs. (ERM or MH)**	**LAMP2**	1.06	0.0049
**RD vs. (ERM or MH)**	**MFAP4**	1.06	0.0049
**RD vs. (ERM or MH)**	**PZP**	1.06	0.0054
**RD vs. (ERM or MH)**	**HABP2**	1.06	0.007
**RD vs. (ERM or MH)**	**CPN2**	1.07	0.0029
**RD vs. (ERM or MH)**	**RBP4**	1.07	0.01
**RD vs. (ERM or MH)**	**TMBIM6**	1.08	0.0026
**RD vs. (ERM or MH)**	**IGFBP2**	1.08	0.0045
**RD vs. (ERM or MH)**	**NELL2**	1.08	0.0045
**RD vs. (ERM or MH)**	**ASAH1**	1.08	0.0053
**RD vs. (ERM or MH)**	**NFASC**	1.09	0.0065
**RD vs. (ERM or MH)**	**RNASE1**	1.09	0.0074
**RD vs. (ERM or MH)**	**IGHV3-13**	1.1	0.0046
**RD vs. (ERM or MH)**	**PYGM**	1.11	0.016
**RD vs. (ERM or MH)**	**APOF**	1.12	0.0023
**RD vs. (ERM or MH)**	**MEGF8**	1.12	0.0045
**RD vs. (ERM or MH)**	**FGA**	1.12	0.021
**RD vs. (ERM or MH)**	**IGHV3-15**	1.13	0.0029
**RD vs. (ERM or MH)**	**IGHV3-35**	1.13	0.0068
**RD vs. (ERM or MH)**	**DPP7**	1.13	0.0094
**RD vs. (ERM or MH)**	**KLKB1**	1.14	0.0035
**RD vs. (ERM or MH)**	**F5**	1.14	0.0043
**RD vs. (ERM or MH)**	**CPAMD8**	1.14	0.0081
**RD vs. (ERM or MH)**	**MINPP1**	1.15	0.0011
**RD vs. (ERM or MH)**	**QPCT**	1.15	0.0015
**RD vs. (ERM or MH)**	**GNS**	1.15	0.003
**RD vs. (ERM or MH)**	**FABP5**	1.15	0.005
**RD vs. (ERM or MH)**	**ATP6AP1**	1.16	0.0019
**RD vs. (ERM or MH)**	**TNC**	1.17	0.0025
**RD vs. (ERM or MH)**	**ECM1**	1.17	0.0038
**RD vs. (ERM or MH)**	**MANBA**	1.18	0.00096
**RD vs. (ERM or MH)**	**IGKV1-5**	1.18	0.0027

Differentially Expressed proteins			
**Comparison**	**SYMBOL**	**logFold**	**p-value**
**RD vs. (ERM or MH)**	**PKM**	1.18	0.004
**RD vs. (ERM or MH)**	**TNR**	1.19	0.0033
**RD vs. (ERM or MH)**	**CLSTN2**	1.19	0.0042
**RD vs. (ERM or MH)**	**C8G**	1.2	0.00089
**RD vs. (ERM or MH)**	**SERPINF1**	1.21	0.00041
**RD vs. (ERM or MH)**	**IGKV3-15**	1.21	0.0015
**RD vs. (ERM or MH)**	**BTD**	1.21	0.003
**RD vs. (ERM or MH)**	**CTSF**	1.22	0.0012
**RD vs. (ERM or MH)**	**CLEC3B**	1.22	0.0045
**RD vs. (ERM or MH)**	**PGLYRP2**	1.23	0.00046
**RD vs. (ERM or MH)**	**SERPINA10**	1.23	0.00056
**RD vs. (ERM or MH)**	**SCG5**	1.23	0.0011
**RD vs. (ERM or MH)**	**SFRP4**	1.23	0.0011
**RD vs. (ERM or MH)**	**DAG1**	1.23	0.0019
**RD vs. (ERM or MH)**	**EPDR1**	1.23	0.0023
**RD vs. (ERM or MH)**	**SIAE**	1.24	0.00037
**RD vs. (ERM or MH)**	**SERPIND1**	1.24	0.00063
**RD vs. (ERM or MH)**	**TPP1**	1.24	0.00092
**RD vs. (ERM or MH)**	**APP**	1.24	0.0011
**RD vs. (ERM or MH)**	**SHBG**	1.24	0.0012
**RD vs. (ERM or MH)**	**PSAP**	1.24	0.0041
**RD vs. (ERM or MH)**	**CTSA**	1.24	0.006
**RD vs. (ERM or MH)**	**PCOLCE**	1.25	0.00095
**RD vs. (ERM or MH)**	**CFH**	1.25	0.0017
**RD vs. (ERM or MH)**	**APOA2**	1.25	0.0046
**RD vs. (ERM or MH)**	**NTM**	1.26	0.00048
**RD vs. (ERM or MH)**	**C8B**	1.26	0.00088
**RD vs. (ERM or MH)**	**ITIH5**	1.26	0.0038
**RD vs. (ERM or MH)**	**CPN1**	1.27	0.00045
**RD vs. (ERM or MH)**	**IGLV1-51**	1.27	0.00045
**RD vs. (ERM or MH)**	**C8A**	1.27	0.00067
**RD vs. (ERM or MH)**	**DNAH10**	1.27	0.0011
**RD vs. (ERM or MH)**	**TIMP1**	1.27	0.0018
**RD vs. (ERM or MH)**	**OAF**	1.27	0.002
**RD vs. (ERM or MH)**	**LRIT1**	1.28	0.0014
**RD vs. (ERM or MH)**	**CRYGS**	1.28	0.0018
**RD vs. (ERM or MH)**	**TIMP2**	1.28	0.0018
**RD vs. (ERM or MH)**	**CHL1**	1.29	0.0022
**RD vs. (ERM or MH)**	**CDH2**	1.29	0.0054

Differentially Expressed proteins			
**Comparison**	**SYMBOL**	**logFold**	**p-value**
**RD vs. (ERM or MH)**	**IGHV3-48**	1.29	0.0078
**RD vs. (ERM or MH)**	**BCAN**	1.3	0.00029
**RD vs. (ERM or MH)**	**SERPINF2**	1.3	0.00035
**RD vs. (ERM or MH)**	**CLSTN3**	1.3	0.0029
**RD vs. (ERM or MH)**	**GSN**	1.3	0.0033
**RD vs. (ERM or MH)**	**CHGA**	1.31	0.00019
**RD vs. (ERM or MH)**	**BTBD16**	1.31	0.00044
**RD vs. (ERM or MH)**	**LUM**	1.31	6.00E-04
**RD vs. (ERM or MH)**	**ZNF33B**	1.33	0.00031
**RD vs. (ERM or MH)**	**CADM2**	1.33	0.00063
**RD vs. (ERM or MH)**	**F9**	1.33	0.0014
**RD vs. (ERM or MH)**	**COL9A2**	1.35	0.00016
**RD vs. (ERM or MH)**	**CRYBA1**	1.35	0.0018
**RD vs. (ERM or MH)**	**TRMT1L**	1.36	0.0024
**RD vs. (ERM or MH)**	**COL1A2**	1.37	0.0047
**RD vs. (ERM or MH)**	**DNASE2**	1.38	0.00011
**RD vs. (ERM or MH)**	**C6**	1.39	0.00013
**RD vs. (ERM or MH)**	**GRIA4**	1.39	0.00023
**RD vs. (ERM or MH)**	**ITIH4**	1.39	0.00042
**RD vs. (ERM or MH)**	**FN1**	1.39	0.0031
**RD vs. (ERM or MH)**	**NRXN3**	1.4	0.00019
**RD vs. (ERM or MH)**	**PAM**	1.4	0.00027
**RD vs. (ERM or MH)**	**PEBP4**	1.4	0.00043
**RD vs. (ERM or MH)**	**C1S**	1.41	0.00014
**RD vs. (ERM or MH)**	**HBB**	1.41	0.027
**RD vs. (ERM or MH)**	**COL18A1**	1.42	3.00E-04
**RD vs. (ERM or MH)**	**VASN**	1.43	0.00014
**RD vs. (ERM or MH)**	**RARRES2**	1.43	0.00026
**RD vs. (ERM or MH)**	**HBD**	1.43	0.0025
**RD vs. (ERM or MH)**	**IGKV6D-41**	1.44	0.00014
**RD vs. (ERM or MH)**	**RAB11FIP1**	1.44	0.00014
**RD vs. (ERM or MH)**	**NEGR1**	1.44	0.00017
**RD vs. (ERM or MH)**	**SERPINA6**	1.45	6.80E-05
**RD vs. (ERM or MH)**	**ZSWIM9**	1.45	0.00012
**RD vs. (ERM or MH)**	**SERPINA5**	1.45	0.00067
**RD vs. (ERM or MH)**	**CRYBB1**	1.45	0.0019
**RD vs. (ERM or MH)**	**ITIH3**	1.46	0.00021
**RD vs. (ERM or MH)**	**CADM1**	1.46	0.00045
**RD vs. (ERM or MH)**	**ALG13**	1.46	0.00052

Differentially Expressed proteins			
**Comparison**	**SYMBOL**	**logFold**	**p-value**
**RD vs. (ERM or MH)**	**CNTN4**	1.46	0.00052
**RD vs. (ERM or MH)**	**GOLIM4**	1.46	0.00052
**RD vs. (ERM or MH)**	**SPOCK2**	1.46	0.00052
**RD vs. (ERM or MH)**	**MYOC**	1.47	0.00054
**RD vs. (ERM or MH)**	**TF**	1.48	4.80E-06
**RD vs. (ERM or MH)**	**FAM3C**	1.48	0.00021
**RD vs. (ERM or MH)**	**ITGA8**	1.48	0.00032
**RD vs. (ERM or MH)**	**CRYAA**	1.48	0.00046
**RD vs. (ERM or MH)**	**LTBP2**	1.48	0.00064
**RD vs. (ERM or MH)**	**PTGDS**	1.49	0.00066
**RD vs. (ERM or MH)**	**APOA1**	1.5	4.80E-05
**RD vs. (ERM or MH)**	**LTF**	1.52	6.10E-05
**RD vs. (ERM or MH)**	**PIK3IP1**	1.52	0.00014
**RD vs. (ERM or MH)**	**IGF2**	1.52	0.00019
**RD vs. (ERM or MH)**	**SPOCK1**	1.52	0.00028
**RD vs. (ERM or MH)**	**CRYBB2**	1.53	0.019
**RD vs. (ERM or MH)**	**SPARCL1**	1.54	2.00E-04
**RD vs. (ERM or MH)**	**B4GAT1**	1.55	4.00E-05
**RD vs. (ERM or MH)**	**CPE**	1.56	9.30E-05
**RD vs. (ERM or MH)**	**ENPP2**	1.56	0.0012
**RD vs. (ERM or MH)**	**NAGA**	1.57	0.00017
**RD vs. (ERM or MH)**	**AMBP**	1.57	0.00019
**RD vs. (ERM or MH)**	**PLG**	1.57	0.00022
**RD vs. (ERM or MH)**	**IGKV2D-24**	1.58	2.90E-05
**RD vs. (ERM or MH)**	**PTPRZ1**	1.58	5.50E-05
**RD vs. (ERM or MH)**	**ATRN**	1.59	1.60E-05
**RD vs. (ERM or MH)**	**CNDP1**	1.61	1.40E-05
**RD vs. (ERM or MH)**	**C1QC**	1.64	1.70E-05
**RD vs. (ERM or MH)**	**AHSG**	1.64	0.00035
**RD vs. (ERM or MH)**	**SELENOP**	1.65	2.40E-05
**RD vs. (ERM or MH)**	**GAA**	1.65	3.80E-05
**RD vs. (ERM or MH)**	**F2**	1.65	7.90E-05
**RD vs. (ERM or MH)**	**IGLV8-61**	1.67	4.10E-05
**RD vs. (ERM or MH)**	**FBLN1**	1.67	4.50E-05
**RD vs. (ERM or MH)**	**APOA4**	1.67	7.00E-04
**RD vs. (ERM or MH)**	**COL2A1**	1.68	6.50E-05
**RD vs. (ERM or MH)**	**IGFBP7**	1.68	0.00014
**RD vs. (ERM or MH)**	**GAS6**	1.69	2.80E-05
**RD vs. (ERM or MH)**	**C2**	1.69	3.80E-05

Differentially Expressed proteins			
Comparison	SYMBOL	logFold	p-value
RD vs. (ERM or MH)	PRNP	1.69	0.00022
RD vs. (ERM or MH)	COL6A1	1.7	4.90E-06
RD vs. (ERM or MH)	CFI	1.7	3.40E-05
RD vs. (ERM or MH)	ABCF1	1.7	0.00017
RD vs. (ERM or MH)	NCAN	1.72	3.80E-05
RD vs. (ERM or MH)	SEZ6	1.74	1.00E-05
RD vs. (ERM or MH)	SEMA7A	1.75	0.00011
RD vs. (ERM or MH)	PCSK1N	1.77	9.00E-06
RD vs. (ERM or MH)	KRT74	1.77	0.00019
RD vs. (ERM or MH)	FRZB	1.78	1.30E-05
RD vs. (ERM or MH)	APLP2	1.78	1.40E-05
RD vs. (ERM or MH)	NCAM1	1.78	4.80E-05
RD vs. (ERM or MH)	FETUB	1.8	2.70E-05
RD vs. (ERM or MH)	WIF1	1.85	1.70E-06
RD vs. (ERM or MH)	IGKV3D-20	1.85	5.20E-06
RD vs. (ERM or MH)	CFHR2	1.88	0.00011
RD vs. (ERM or MH)	IGHV3-43D	1.91	0.00011
RD vs. (ERM or MH)	SERPINI1	1.92	6.20E-07
RD vs. (ERM or MH)	SPP1	1.94	4.10E-05
RD vs. (ERM or MH)	SPON1	1.95	4.90E-07
RD vs. (ERM or MH)	NPC2	1.95	8.30E-06
RD vs. (ERM or MH)	APOC3	1.96	9.60E-07
RD vs. (ERM or MH)	IGLL5	1.96	3.70E-05
RD vs. (ERM or MH)	APOD	1.96	0.00013
RD vs. (ERM or MH)	ABI3BP	2.04	1.20E-06
RD vs. (ERM or MH)	APOE	2.05	7.00E-06
RD vs. (ERM or MH)	NRCAM	2.07	2.60E-07
RD vs. (ERM or MH)	AGRN	2.13	8.90E-05
RD vs. (ERM or MH)	EFEMP1	2.14	1.00E-06
RD vs. (ERM or MH)	FSTL5	2.16	5.30E-07
RD vs. (ERM or MH)	IGHV3-64D	2.16	9.20E-06
RD vs. (ERM or MH)	CLU	2.17	1.30E-06
RD vs. (ERM or MH)	APLP1	2.19	1.50E-07
RD vs. (ERM or MH)	HSPG2	2.2	2.10E-07
RD vs. (ERM or MH)	FBN1	2.2	4.40E-05
RD vs. (ERM or MH)	CLSTN1	2.32	8.20E-08
RD vs. (ERM or MH)	QSOX1	2.33	2.50E-08
RD vs. (ERM or MH)	VCAN	2.47	9.40E-08
RD vs. (ERM or MH)	CST3	2.69	8.10E-08

Differentially Expressed proteins			
**Comparison**	**SYMBOL**	**logFold**	**p-value**
**RD vs. (ERM or MH)**	**DKK3**	3.12	1.40E-08
**RD vs. (ERM or MH)**	**C4B**	3.66	1.50E-06
**Mild PVR vs. No PVR**	**KRT6A**	-6.79	1.80E-08
**Mild PVR vs. No PVR**	**KRT6B**	-6.77	1.60E-09
**Mild PVR vs. No PVR**	**KRT5**	-6.77	2.50E-08
**Mild PVR vs. No PVR**	**KRT16**	-6.06	4.50E-08
**Mild PVR vs. No PVR**	**KRT77**	-5.67	5.40E-09
**Mild PVR vs. No PVR**	**IGHG2**	-5.44	1.10E-07
**Mild PVR vs. No PVR**	**KRT17**	-5.43	1.00E-08
**Mild PVR vs. No PVR**	**KRT25**	-5.17	1.20E-08
**Mild PVR vs. No PVR**	**KRT71**	-5.15	5.90E-09
**Mild PVR vs. No PVR**	**KRT9**	-4.9	3.40E-06
**Mild PVR vs. No PVR**	**KRT1**	-4.78	5.40E-07
**Mild PVR vs. No PVR**	**KRT14**	-4.41	2.30E-06
**Mild PVR vs. No PVR**	**KRT2**	-4.32	1.70E-06
**Mild PVR vs. No PVR**	**KRT73**	-4.07	7.40E-07
**Mild PVR vs. No PVR**	**KRT10**	-4.07	1.50E-05
**Mild PVR vs. No PVR**	**KRT78**	-3.5	8.00E-06
**Mild PVR vs. No PVR**	**BAX**	-3.46	1.20E-05
**Mild PVR vs. No PVR**	**DSP**	-3.46	6.90E-05
**Mild PVR vs. No PVR**	**PRSS1**	-3.26	3.80E-06
**Mild PVR vs. No PVR**	**KRT27**	-3.19	0.0048
**Mild PVR vs. No PVR**	**KRT28**	-2.88	0.0018
**Mild PVR vs. No PVR**	**IGLL1**	-2.79	0.0099
**Mild PVR vs. No PVR**	**IGLC7**	-2.78	0.0019
**Mild PVR vs. No PVR**	**IGKV4-1**	-2.52	1.10E-05
**Mild PVR vs. No PVR**	**PRSS3**	-2.52	2.60E-05
**Mild PVR vs. No PVR**	**POTEF**	-2.52	6.60E-05
**Mild PVR vs. No PVR**	**FLG2**	-2.52	0.00011
**Mild PVR vs. No PVR**	**TADA2B**	-2.52	0.00022
**Mild PVR vs. No PVR**	**KRT7**	-2.52	0.0024
**Mild PVR vs. No PVR**	**KPRP**	-2.34	0.0046
**Mild PVR vs. No PVR**	**NT5C3B**	-2.17	0.013
**Mild PVR vs. No PVR**	**ANXA2**	-2.1	0.0028
**Mild PVR vs. No PVR**	**DCD**	-2.1	0.0028
**Mild PVR vs. No PVR**	**IGHV3-74**	-2.09	0.013
**Mild PVR vs. No PVR**	**IGHG4**	-2.08	0.0051
**Mild PVR vs. No PVR**	**SERPINB12**	-1.73	0.0081
**Mild PVR vs. No PVR**	**C8orf37**	-1.73	0.0085

Differentially Expressed proteins			
**Comparison**	**SYMBOL**	**logFold**	**p-value**
**Mild PVR vs. No PVR**	**CRB1**	-1.73	0.0085
**Mild PVR vs. No PVR**	**S100A9**	-1.72	0.0087
**Mild PVR vs. No PVR**	**IGHV1OR15-1**	-1.72	0.009
**Mild PVR vs. No PVR**	**IGLV1-47**	-1.7	0.0022
**Mild PVR vs. No PVR**	**SERPINA3**	1.24	0.008
**Mild PVR vs. No PVR**	**SERPINA7**	1.4	0.0042
**Mild PVR vs. No PVR**	**SERPINI1**	1.4	0.0061
**Mild PVR vs. No PVR**	**CNDP1**	1.4	0.0066
**Mild PVR vs. No PVR**	**NRXN3**	1.4	0.011
**Mild PVR vs. No PVR**	**TGFBI**	1.4	0.011
**Mild PVR vs. No PVR**	**PCSK1N**	1.4	0.011
**Mild PVR vs. No PVR**	**MACF1**	1.4	0.011
**Mild PVR vs. No PVR**	**TPP1**	1.4	0.013
**Mild PVR vs. No PVR**	**ATP6AP1**	1.4	0.014
**Mild PVR vs. No PVR**	**HSPA5**	1.4	0.015
**Mild PVR vs. No PVR**	**CFB**	1.43	0.012
**Mild PVR vs. No PVR**	**RTBDN**	1.44	0.011
**Mild PVR vs. No PVR**	**FAM3C**	1.44	0.013
**Mild PVR vs. No PVR**	**PROS1**	1.44	0.015
**Mild PVR vs. No PVR**	**MMP2**	1.44	0.015
**Mild PVR vs. No PVR**	**PDE6A**	1.46	0.014
**Mild PVR vs. No PVR**	**GC**	1.5	0.014
**Mild PVR vs. No PVR**	**CHI3L1**	1.59	0.015
**Mild PVR vs. No PVR**	**COL6A1**	1.64	0.002
**Mild PVR vs. No PVR**	**WIF1**	1.64	0.0025
**Mild PVR vs. No PVR**	**C1S**	1.64	0.0032
**Mild PVR vs. No PVR**	**C8G**	1.64	0.0033
**Mild PVR vs. No PVR**	**LUM**	1.64	0.0043
**Mild PVR vs. No PVR**	**APP**	1.64	0.0046
**Mild PVR vs. No PVR**	**CRTAC1**	1.64	0.0049
**Mild PVR vs. No PVR**	**CPQ**	1.64	0.0057
**Mild PVR vs. No PVR**	**TPI1**	1.64	0.0076
**Mild PVR vs. No PVR**	**SPOCK1**	1.64	0.0078
**Mild PVR vs. No PVR**	**F12**	1.64	0.008
**Mild PVR vs. No PVR**	**LSAMP**	1.64	0.0081
**Mild PVR vs. No PVR**	**ECM1**	1.64	0.0095
**Mild PVR vs. No PVR**	**GOLM1**	1.64	0.012
**Mild PVR vs. No PVR**	**LRG1**	1.71	0.0044
**Mild PVR vs. No PVR**	**PGLYRP2**	1.82	0.00083

Differentially Expressed proteins			
**Comparison**	**SYMBOL**	**logFold**	**p-value**
**Mild PVR vs. No PVR**	**C8A**	1.82	0.0016
**Mild PVR vs. No PVR**	**CFHR1**	1.82	0.0017
**Mild PVR vs. No PVR**	**C1QB**	1.82	0.002
**Mild PVR vs. No PVR**	**GNAT1**	1.82	0.0022
**Mild PVR vs. No PVR**	**CDH2**	1.82	0.011
**Mild PVR vs. No PVR**	**RBP3**	1.83	0.0028
**Mild PVR vs. No PVR**	**AFM**	1.85	0.0021
**Mild PVR vs. No PVR**	**C6**	1.88	7.00E-04
**Mild PVR vs. No PVR**	**FRZB**	1.88	0.0016
**Mild PVR vs. No PVR**	**COL18A1**	1.88	0.0018
**Mild PVR vs. No PVR**	**GNB1**	1.88	0.0021
**Mild PVR vs. No PVR**	**LRP2**	1.88	0.0026
**Mild PVR vs. No PVR**	**IGFBP7**	1.88	0.0041
**Mild PVR vs. No PVR**	**C7**	1.88	0.0064
**Mild PVR vs. No PVR**	**SOD3**	1.88	0.0069
**Mild PVR vs. No PVR**	**B2M**	1.88	0.0078
**Mild PVR vs. No PVR**	**APOE**	1.92	0.0024
**Mild PVR vs. No PVR**	**SERPINF2**	1.96	0.00053
**Mild PVR vs. No PVR**	**SERPIND1**	1.96	0.00057
**Mild PVR vs. No PVR**	**RS1**	1.96	0.00097
**Mild PVR vs. No PVR**	**FBLN1**	1.96	0.0014
**Mild PVR vs. No PVR**	**CFHR2**	1.96	0.006
**Mild PVR vs. No PVR**	**SERPING1**	2.02	9.40E-05
**Mild PVR vs. No PVR**	**TF**	2.04	3.50E-05
**Mild PVR vs. No PVR**	**ATRN**	2.06	0.00022
**Mild PVR vs. No PVR**	**NRCAM**	2.06	0.00024
**Mild PVR vs. No PVR**	**C1R**	2.06	0.00035
**Mild PVR vs. No PVR**	**RHO**	2.06	0.00051
**Mild PVR vs. No PVR**	**VTN**	2.06	0.00052
**Mild PVR vs. No PVR**	**PON1**	2.06	0.00082
**Mild PVR vs. No PVR**	**RBP4**	2.06	0.0023
**Mild PVR vs. No PVR**	**APOD**	2.06	0.0063
**Mild PVR vs. No PVR**	**PTGDS**	2.16	0.00095
**Mild PVR vs. No PVR**	**GSN**	2.18	0.0018
**Mild PVR vs. No PVR**	**SPON1**	2.19	8.50E-05
**Mild PVR vs. No PVR**	**ABI3BP**	2.21	0.00026
**Mild PVR vs. No PVR**	**CPE**	2.21	0.00032
**Mild PVR vs. No PVR**	**SERPINA4**	2.21	0.0014
**Mild PVR vs. No PVR**	**CPVL**	2.21	0.0015

Differentially Expressed proteins			
**Comparison**	**SYMBOL**	**logFold**	**p-value**
**Mild PVR vs. No PVR**	**SERPINA6**	2.24	9.30E-05
**Mild PVR vs. No PVR**	**C2**	2.24	0.00036
**Mild PVR vs. No PVR**	**VCAN**	2.32	0.00023
**Mild PVR vs. No PVR**	**B4GAT1**	2.38	6.30E-05
**Mild PVR vs. No PVR**	**NPC2**	2.38	0.00025
**Mild PVR vs. No PVR**	**C5**	2.52	0.00014
**Mild PVR vs. No PVR**	**ACTB**	2.53	0.00037
**Mild PVR vs. No PVR**	**AHSG**	2.6	0.00023
**Mild PVR vs. No PVR**	**ITIH4**	2.64	4.30E-05
**Mild PVR vs. No PVR**	**CPAMD8**	2.65	0.00026
**Mild PVR vs. No PVR**	**FN1**	2.68	0.00051
**Mild PVR vs. No PVR**	**CDHR1**	2.71	0.00077
**Mild PVR vs. No PVR**	**HSPG2**	2.75	9.90E-06
**Mild PVR vs. No PVR**	**COL2A1**	2.75	3.90E-05
**Mild PVR vs. No PVR**	**C4B**	2.75	0.0085
**Mild PVR vs. No PVR**	**PKM**	2.86	4.60E-05
**Mild PVR vs. No PVR**	**FSTL5**	2.96	4.50E-06
**Mild PVR vs. No PVR**	**AMBP**	2.97	1.30E-05
**Mild PVR vs. No PVR**	**FGB**	2.97	5.10E-05
**Mild PVR vs. No PVR**	**SEMA7A**	3.06	2.20E-05
**Mild PVR vs. No PVR**	**CLU**	3.1	6.40E-06
**Mild PVR vs. No PVR**	**PLG**	3.15	8.30E-06
**Mild PVR vs. No PVR**	**CTSD**	3.16	1.00E-05
**Mild PVR vs. No PVR**	**APOA4**	3.18	5.40E-05
**Mild PVR vs. No PVR**	**APOH**	3.23	8.10E-06
**Mild PVR vs. No PVR**	**IGLL5**	3.31	9.60E-06
**Mild PVR vs. No PVR**	**KNG1**	3.39	1.70E-06
**Mild PVR vs. No PVR**	**CLSTN1**	3.42	2.00E-07
**Mild PVR vs. No PVR**	**SPP1**	3.45	4.60E-06
**Mild PVR vs. No PVR**	**ENPP2**	3.46	1.30E-05
**Mild PVR vs. No PVR**	**F2**	3.52	6.70E-07
**Mild PVR vs. No PVR**	**APLP2**	3.55	1.90E-07
**Mild PVR vs. No PVR**	**CFI**	3.65	1.60E-07
**Mild PVR vs. No PVR**	**EFEMP1**	3.84	5.00E-08
**Mild PVR vs. No PVR**	**CST3**	3.87	2.50E-07
**Mild PVR vs. No PVR**	**DKK3**	4.34	9.10E-08
**Mild PVR vs. No PVR**	**CFH**	4.47	5.60E-09
**Severe PVR vs. No PVR**	**IGHG2**	-6.17	4.10E-08
**Severe PVR vs. No PVR**	**IGLL1**	-3.85	0.0025

Differentially Expressed proteins			
**Comparison**	**SYMBOL**	**logFold**	**p-value**
**Severe PVR vs. No PVR**	**KRT5**	-3.58	0.0021
**Severe PVR vs. No PVR**	**KRT77**	-2.62	0.0033
**Severe PVR vs. No PVR**	**NT5C3B**	-2.5	0.013
**Severe PVR vs. No PVR**	**IGKV4-1**	-2.41	0.00016
**Severe PVR vs. No PVR**	**APOB**	-2.31	0.0079
**Severe PVR vs. No PVR**	**KRT71**	-2.05	0.0088
**Severe PVR vs. No PVR**	**SERPINF1**	1.44	0.011
**Severe PVR vs. No PVR**	**MINPP1**	1.52	0.011
**Severe PVR vs. No PVR**	**SELENOP**	1.52	0.014
**Severe PVR vs. No PVR**	**HSP90AA1**	1.52	0.014
**Severe PVR vs. No PVR**	**C1QB**	1.66	0.0098
**Severe PVR vs. No PVR**	**IGFBP5**	1.68	0.0085
**Severe PVR vs. No PVR**	**RLBP1**	1.69	0.012
**Severe PVR vs. No PVR**	**CHGA**	1.71	0.0037
**Severe PVR vs. No PVR**	**CTSZ**	1.71	0.0045
**Severe PVR vs. No PVR**	**MANBA**	1.71	0.0052
**Severe PVR vs. No PVR**	**C8G**	1.71	0.0059
**Severe PVR vs. No PVR**	**HSPA13**	1.71	0.0063
**Severe PVR vs. No PVR**	**SEMA4B**	1.71	0.0063
**Severe PVR vs. No PVR**	**AGA**	1.71	0.0076
**Severe PVR vs. No PVR**	**DNER**	1.71	0.0076
**Severe PVR vs. No PVR**	**OLFM1**	1.71	0.0076
**Severe PVR vs. No PVR**	**SEMA3F**	1.71	0.0076
**Severe PVR vs. No PVR**	**GNS**	1.71	0.01
**Severe PVR vs. No PVR**	**IGF2**	1.72	0.011
**Severe PVR vs. No PVR**	**APLP1**	1.75	0.0039
**Severe PVR vs. No PVR**	**MYOC**	1.78	0.012
**Severe PVR vs. No PVR**	**PEBP1**	1.82	0.013
**Severe PVR vs. No PVR**	**PON1**	1.83	0.0061
**Severe PVR vs. No PVR**	**IGHV5-10-1**	1.85	0.01
**Severe PVR vs. No PVR**	**DNASE2**	1.87	0.0018
**Severe PVR vs. No PVR**	**ENO3**	1.87	0.0026
**Severe PVR vs. No PVR**	**IL6ST**	1.87	0.0028
**Severe PVR vs. No PVR**	**MDH1**	1.87	0.0028
**Severe PVR vs. No PVR**	**PTPRG**	1.87	0.0028
**Severe PVR vs. No PVR**	**CLN5**	1.87	0.0031
**Severe PVR vs. No PVR**	**MFGE8**	1.87	0.0031
**Severe PVR vs. No PVR**	**IDS**	1.87	0.0035
**Severe PVR vs. No PVR**	**MAN1A1**	1.87	0.0035

Differentially Expressed proteins			
**Comparison**	**SYMBOL**	**logFold**	**p-value**
**Severe PVR vs. No PVR**	**SPARC**	1.87	0.006
**Severe PVR vs. No PVR**	**SCG2**	1.87	0.0074
**Severe PVR vs. No PVR**	**SCG3**	1.87	0.0078
**Severe PVR vs. No PVR**	**ITIH5**	1.87	0.012
**Severe PVR vs. No PVR**	**CLEC3B**	1.88	0.013
**Severe PVR vs. No PVR**	**FSTL1**	1.89	0.0021
**Severe PVR vs. No PVR**	**C2**	1.89	0.0049
**Severe PVR vs. No PVR**	**ALDOC**	1.91	0.0038
**Severe PVR vs. No PVR**	**SOD3**	1.92	0.013
**Severe PVR vs. No PVR**	**F5**	1.93	0.0063
**Severe PVR vs. No PVR**	**PRDX1**	1.94	0.0063
**Severe PVR vs. No PVR**	**ENO2**	1.94	0.0081
**Severe PVR vs. No PVR**	**FBLN1**	1.98	0.0034
**Severe PVR vs. No PVR**	**CLSTN3**	1.98	0.0085
**Severe PVR vs. No PVR**	**C8A**	1.99	0.0021
**Severe PVR vs. No PVR**	**COL9A1**	2.01	0.0024
**Severe PVR vs. No PVR**	**ATP6AP1**	2.01	0.0025
**Severe PVR vs. No PVR**	**NCAM1**	2.03	0.0041
**Severe PVR vs. No PVR**	**CHL1**	2.04	0.0058
**Severe PVR vs. No PVR**	**RNASE1**	2.05	0.005
**Severe PVR vs. No PVR**	**EFEMP1**	2.07	0.0022
**Severe PVR vs. No PVR**	**SIAE**	2.08	0.00063
**Severe PVR vs. No PVR**	**COL9A2**	2.08	0.00073
**Severe PVR vs. No PVR**	**NTM**	2.08	0.00098
**Severe PVR vs. No PVR**	**PTPRZ1**	2.08	0.0016
**Severe PVR vs. No PVR**	**FAM3C**	2.09	0.0021
**Severe PVR vs. No PVR**	**CKB**	2.1	0.0016
**Severe PVR vs. No PVR**	**NELL2**	2.1	0.0021
**Severe PVR vs. No PVR**	**CADM1**	2.1	0.0029
**Severe PVR vs. No PVR**	**CD59**	2.13	0.0014
**Severe PVR vs. No PVR**	**MMP2**	2.14	0.0022
**Severe PVR vs. No PVR**	**PIK3IP1**	2.15	0.0017
**Severe PVR vs. No PVR**	**PAPPA2**	2.15	0.0037
**Severe PVR vs. No PVR**	**ATRN**	2.17	0.00047
**Severe PVR vs. No PVR**	**PCSK1N**	2.17	0.00093
**Severe PVR vs. No PVR**	**RTBDN**	2.19	0.0011
**Severe PVR vs. No PVR**	**TNR**	2.23	0.0021
**Severe PVR vs. No PVR**	**HSPA1B**	2.24	0.00056
**Severe PVR vs. No PVR**	**MST1**	2.24	0.0011

Differentially Expressed proteins			
**Comparison**	**SYMBOL**	**logFold**	**p-value**
**Severe PVR vs. No PVR**	**IGHV6-1**	2.25	0.001
**Severe PVR vs. No PVR**	**IMPG1**	2.25	0.013
**Severe PVR vs. No PVR**	**SERPINI1**	2.26	0.00023
**Severe PVR vs. No PVR**	**CNDP1**	2.26	0.00026
**Severe PVR vs. No PVR**	**QSOX1**	2.26	0.00026
**Severe PVR vs. No PVR**	**HEXB**	2.26	0.00068
**Severe PVR vs. No PVR**	**PRKCSH**	2.26	0.00088
**Severe PVR vs. No PVR**	**CTSL**	2.26	0.0012
**Severe PVR vs. No PVR**	**RNASET2**	2.26	0.0017
**Severe PVR vs. No PVR**	**ECM1**	2.27	0.0021
**Severe PVR vs. No PVR**	**CPAMD8**	2.29	0.0032
**Severe PVR vs. No PVR**	**COL11A1**	2.29	0.0042
**Severe PVR vs. No PVR**	**CFHR2**	2.32	0.0043
**Severe PVR vs. No PVR**	**TPI1**	2.34	0.0012
**Severe PVR vs. No PVR**	**IGFBP7**	2.34	0.0019
**Severe PVR vs. No PVR**	**NRXN3**	2.39	0.00033
**Severe PVR vs. No PVR**	**TPP1**	2.39	0.00043
**Severe PVR vs. No PVR**	**GOLM1**	2.39	0.0018
**Severe PVR vs. No PVR**	**APOE**	2.4	0.0012
**Severe PVR vs. No PVR**	**GPX3**	2.42	0.00072
**Severe PVR vs. No PVR**	**COL6A1**	2.43	0.00012
**Severe PVR vs. No PVR**	**CRTAC1**	2.43	0.00043
**Severe PVR vs. No PVR**	**CPQ**	2.43	0.00053
**Severe PVR vs. No PVR**	**LRP1**	2.43	0.00075
**Severe PVR vs. No PVR**	**ENPP2**	2.47	0.0029
**Severe PVR vs. No PVR**	**PLD3**	2.48	0.00085
**Severe PVR vs. No PVR**	**FUCA2**	2.48	0.00088
**Severe PVR vs. No PVR**	**CLU**	2.49	0.00052
**Severe PVR vs. No PVR**	**CFI**	2.5	0.00032
**Severe PVR vs. No PVR**	**NCAN**	2.5	0.00036
**Severe PVR vs. No PVR**	**APP**	2.51	0.00028
**Severe PVR vs. No PVR**	**MACF1**	2.62	0.00012
**Severe PVR vs. No PVR**	**HSPA5**	2.62	0.00021
**Severe PVR vs. No PVR**	**FSTL4**	2.62	0.00083
**Severe PVR vs. No PVR**	**SPOCK1**	2.63	0.00038
**Severe PVR vs. No PVR**	**COL18A1**	2.67	0.00018
**Severe PVR vs. No PVR**	**PDE6A**	2.68	0.00023
**Severe PVR vs. No PVR**	**LSAMP**	2.7	0.00029
**Severe PVR vs. No PVR**	**PTGDS**	2.71	0.00042

Differentially Expressed proteins			
**Comparison**	**SYMBOL**	**logFold**	**p-value**
**Severe PVR vs. No PVR**	**CDH2**	2.73	0.0013
**Severe PVR vs. No PVR**	**FRZB**	2.75	0.00011
**Severe PVR vs. No PVR**	**IMPG2**	2.79	0.0011
**Severe PVR vs. No PVR**	**GNB1**	2.82	0.00011
**Severe PVR vs. No PVR**	**LRP2**	2.82	0.00015
**Severe PVR vs. No PVR**	**WIF1**	2.85	2.00E-05
**Severe PVR vs. No PVR**	**NPC2**	2.87	0.00012
**Severe PVR vs. No PVR**	**ACTB**	2.89	0.00035
**Severe PVR vs. No PVR**	**RS1**	2.95	3.80E-05
**Severe PVR vs. No PVR**	**SPON1**	2.97	7.50E-06
**Severe PVR vs. No PVR**	**NRCAM**	2.97	1.10E-05
**Severe PVR vs. No PVR**	**B4GAT1**	2.97	1.80E-05
**Severe PVR vs. No PVR**	**CPVL**	3	0.00023
**Severe PVR vs. No PVR**	**GNAT1**	3.04	2.90E-05
**Severe PVR vs. No PVR**	**CDHR1**	3.14	0.00063
**Severe PVR vs. No PVR**	**ABI3BP**	3.2	1.10E-05
**Severe PVR vs. No PVR**	**CPE**	3.2	1.50E-05
**Severe PVR vs. No PVR**	**RHO**	3.28	6.40E-06
**Severe PVR vs. No PVR**	**VCAN**	3.3	1.20E-05
**Severe PVR vs. No PVR**	**RBP3**	3.35	2.30E-05
**Severe PVR vs. No PVR**	**PKM**	3.45	2.20E-05
**Severe PVR vs. No PVR**	**IGLL5**	3.46	3.10E-05
**Severe PVR vs. No PVR**	**CLSTN1**	3.5	1.20E-06
**Severe PVR vs. No PVR**	**FSTL5**	3.68	1.10E-06
**Severe PVR vs. No PVR**	**SPP1**	3.72	1.10E-05
**Severe PVR vs. No PVR**	**HSPG2**	3.74	5.90E-07
**Severe PVR vs. No PVR**	**SEMA7A**	3.75	7.90E-06
**Severe PVR vs. No PVR**	**COL2A1**	3.82	2.10E-06
**Severe PVR vs. No PVR**	**DKK3**	3.87	6.10E-06
**Severe PVR vs. No PVR**	**CST3**	4.27	5.10E-07
**Severe PVR vs. No PVR**	**APLP2**	4.54	2.50E-08
**Severe PVR vs. Mild PVR**	**CFH**	-3.21	2.30E-06
**Severe PVR vs. Mild PVR**	**FGB**	-3.02	1.70E-05
**Severe PVR vs. Mild PVR**	**ITIH4**	-2.91	3.60E-06
**Severe PVR vs. Mild PVR**	**APOH**	-2.84	2.50E-05
**Severe PVR vs. Mild PVR**	**KNG1**	-2.75	2.40E-05
**Severe PVR vs. Mild PVR**	**FN1**	-2.75	0.00018
**Severe PVR vs. Mild PVR**	**C5**	-2.63	3.50E-05
**Severe PVR vs. Mild PVR**	**F2**	-2.55	5.90E-05

Differentially Expressed proteins			
**Comparison**	**SYMBOL**	**logFold**	**p-value**
**Severe PVR vs. Mild PVR**	**IGHG3**	-2.47	0.0056
**Severe PVR vs. Mild PVR**	**APOA4**	-2.35	0.0012
**Severe PVR vs. Mild PVR**	**CHI3L1**	-2.23	0.00039
**Severe PVR vs. Mild PVR**	**PLG**	-2.19	0.00055
**Severe PVR vs. Mild PVR**	**GSN**	-2.13	0.0013
**Severe PVR vs. Mild PVR**	**SERPINF2**	-1.97	0.00028
**Severe PVR vs. Mild PVR**	**RBP4**	-1.94	0.0024
**Severe PVR vs. Mild PVR**	**CFB**	-1.92	0.00046
**Severe PVR vs. Mild PVR**	**AHSG**	-1.91	0.0035
**Severe PVR vs. Mild PVR**	**AFM**	-1.83	0.0013
**Severe PVR vs. Mild PVR**	**PGLYRP2**	-1.78	6.00E-04
**Severe PVR vs. Mild PVR**	**EFEMP1**	-1.77	0.0021
**Severe PVR vs. Mild PVR**	**SERPINA7**	-1.73	4.00E-04
**Severe PVR vs. Mild PVR**	**IGHA1**	-1.64	0.0032
**Severe PVR vs. Mild PVR**	**AMBP**	-1.61	0.0057
**Severe PVR vs. Mild PVR**	**SERPIND1**	-1.56	0.0027
**Severe PVR vs. Mild PVR**	**FCGBP**	-1.44	0.0057
**Severe PVR vs. Mild PVR**	**SERPINB12**	1.79	0.0043
**Severe PVR vs. Mild PVR**	**S100A9**	1.79	0.0047
**Severe PVR vs. Mild PVR**	**POTEF**	1.81	0.0012
**Severe PVR vs. Mild PVR**	**TADA2B**	1.82	0.0028
**Severe PVR vs. Mild PVR**	**IGLV1-47**	1.99	0.00025
**Severe PVR vs. Mild PVR**	**FLG2**	2.06	0.00057
**Severe PVR vs. Mild PVR**	**UBA52**	2.14	0.00043
**Severe PVR vs. Mild PVR**	**DSP**	2.2	0.0035
**Severe PVR vs. Mild PVR**	**ANXA2**	2.39	0.00051
**Severe PVR vs. Mild PVR**	**DCD**	2.39	0.00051
**Severe PVR vs. Mild PVR**	**KRT14**	2.4	0.0024
**Severe PVR vs. Mild PVR**	**KRT10**	2.41	0.0012
**Severe PVR vs. Mild PVR**	**KRT78**	2.63	0.00014
**Severe PVR vs. Mild PVR**	**KRT2**	2.67	0.00016
**Severe PVR vs. Mild PVR**	**KRT1**	2.72	0.00016
**Severe PVR vs. Mild PVR**	**PRSS3**	2.8	2.20E-06
**Severe PVR vs. Mild PVR**	**KRT7**	2.8	5.00E-04
**Severe PVR vs. Mild PVR**	**KRT77**	3.06	9.80E-05
**Severe PVR vs. Mild PVR**	**IGLC7**	3.07	0.00043
**Severe PVR vs. Mild PVR**	**KRT71**	3.1	1.90E-05
**Severe PVR vs. Mild PVR**	**KRT28**	3.17	0.00042
**Severe PVR vs. Mild PVR**	**KRT9**	3.2	0.00019

Differentially Expressed proteins			
**Comparison**	**SYMBOL**	**logFold**	**p-value**
**Severe PVR vs. Mild PVR**	**KRT5**	3.2	0.00078
**Severe PVR vs. Mild PVR**	**PRSS1**	3.22	2.00E-06
**Severe PVR vs. Mild PVR**	**BAX**	3.42	6.80E-06
**Severe PVR vs. Mild PVR**	**KRT16**	3.75	6.00E-05
**Severe PVR vs. Mild PVR**	**KRT73**	3.85	8.40E-07
**Severe PVR vs. Mild PVR**	**KRT6A**	4.14	4.20E-05
**Severe PVR vs. Mild PVR**	**KRT6B**	4.61	1.10E-06
**Severe PVR vs. Mild PVR**	**KRT25**	4.89	1.30E-08
**Severe PVR vs. Mild PVR**	**KRT17**	5.09	1.30E-08
**PVR vs. No PVR**	**IGHG2**	-5.80	1E-08
**PVR vs. No PVR**	**KRT5**	-5.18	2.3E-06
**PVR vs. No PVR**	**KRT6A**	-4.72	8.6E-06
**PVR vs. No PVR**	**KRT6B**	-4.46	0.000003
**PVR vs. No PVR**	**KRT16**	-4.19	0.000016
**PVR vs. No PVR**	**KRT77**	-4.15	1.2E-06
**PVR vs. No PVR**	**KRT71**	-3.60	2.2E-06
**PVR vs. No PVR**	**KRT1**	-3.42	0.00006
**PVR vs. No PVR**	**IGLL1**	-3.32	0.002
**PVR vs. No PVR**	**KRT9**	-3.30	0.00052
**PVR vs. No PVR**	**KRT14**	-3.21	0.00014
**PVR vs. No PVR**	**KRT2**	-2.99	0.00022
**PVR vs. No PVR**	**KRT17**	-2.89	0.00016
**PVR vs. No PVR**	**KRT10**	-2.86	0.00077
**PVR vs. No PVR**	**KRT25**	-2.73	0.00018
**PVR vs. No PVR**	**IGKV4-1**	-2.47	0.000011
**PVR vs. No PVR**	**DSP**	-2.35	0.0025
**PVR vs. No PVR**	**NT5C3B**	-2.34	0.0062
**PVR vs. No PVR**	**KRT78**	-2.18	0.0014
**PVR vs. No PVR**	**KRT73**	-2.14	0.0017
**PVR vs. No PVR**	**IGHG4**	-1.93	0.0065
**PVR vs. No PVR**	**BAX**	-1.74	0.0097
**PVR vs. No PVR**	**PRSS1**	-1.64	0.0058
**PVR vs. No PVR**	**POTEF**	-1.61	0.0046
**PVR vs. No PVR**	**TADA2B**	-1.61	0.0095
**PVR vs. No PVR**	**FLG2**	-1.49	0.011
**PVR vs. No PVR**	**SERPINF1**	1.18	0.012
**PVR vs. No PVR**	**CHGA**	1.18	0.014
**PVR vs. No PVR**	**CTSZ**	1.18	0.016
**PVR vs. No PVR**	**MANBA**	1.18	0.018

Differentially Expressed proteins			
**Comparison**	**SYMBOL**	**logFold**	**p-value**
**PVR vs. No PVR**	**DNASE2**	1.26	0.0097
**PVR vs. No PVR**	**ENO3**	1.26	0.013
**PVR vs. No PVR**	**IL6ST**	1.26	0.013
**PVR vs. No PVR**	**MDH1**	1.26	0.013
**PVR vs. No PVR**	**PTPRG**	1.26	0.013
**PVR vs. No PVR**	**CLN5**	1.26	0.014
**PVR vs. No PVR**	**MFGE8**	1.26	0.014
**PVR vs. No PVR**	**IDS**	1.26	0.016
**PVR vs. No PVR**	**MAN1A1**	1.26	0.016
**PVR vs. No PVR**	**C6**	1.28	0.012
**PVR vs. No PVR**	**CFHR1**	1.31	0.015
**PVR vs. No PVR**	**C1S**	1.34	0.011
**PVR vs. No PVR**	**PROS1**	1.35	0.017
**PVR vs. No PVR**	**RLBP1**	1.37	0.016
**PVR vs. No PVR**	**IGF2**	1.37	0.015
**PVR vs. No PVR**	**APLP1**	1.40	0.0058
**PVR vs. No PVR**	**FSTL1**	1.45	0.0043
**PVR vs. No PVR**	**TIMP1**	1.47	0.014
**PVR vs. No PVR**	**LRG1**	1.47	0.01
**PVR vs. No PVR**	**ALDOC**	1.47	0.007
**PVR vs. No PVR**	**F5**	1.47	0.012
**PVR vs. No PVR**	**SERPING1**	1.48	0.0015
**PVR vs. No PVR**	**C1R**	1.49	0.0049
**PVR vs. No PVR**	**TF**	1.49	0.00059
**PVR vs. No PVR**	**CLSTN3**	1.51	0.016
**PVR vs. No PVR**	**COL9A1**	1.51	0.0056
**PVR vs. No PVR**	**NCAM1**	1.53	0.0087
**PVR vs. No PVR**	**SIAE**	1.55	0.002
**PVR vs. No PVR**	**COL9A2**	1.55	0.0022
**PVR vs. No PVR**	**NTM**	1.55	0.0028
**PVR vs. No PVR**	**PTPRZ1**	1.55	0.0044
**PVR vs. No PVR**	**PEBP1**	1.55	0.012
**PVR vs. No PVR**	**CKB**	1.57	0.0042
**PVR vs. No PVR**	**NELL2**	1.57	0.0053
**PVR vs. No PVR**	**CADM1**	1.57	0.007
**PVR vs. No PVR**	**VTN**	1.57	0.0049
**PVR vs. No PVR**	**F12**	1.58	0.0076
**PVR vs. No PVR**	**SERPINA6**	1.59	0.0024
**PVR vs. No PVR**	**PRDX1**	1.61	0.0068

Differentially Expressed proteins			
**Comparison**	**SYMBOL**	**logFold**	**p-value**
**PVR vs. No PVR**	**MYOC**	1.61	0.008
**PVR vs. No PVR**	**IGHV5-10-1**	1.62	0.0081
**PVR vs. No PVR**	**HSPA1B**	1.63	0.0022
**PVR vs. No PVR**	**MST1**	1.63	0.0037
**PVR vs. No PVR**	**C7**	1.64	0.012
**PVR vs. No PVR**	**QSOX1**	1.65	0.0011
**PVR vs. No PVR**	**HEXB**	1.65	0.0025
**PVR vs. No PVR**	**PRKCSH**	1.65	0.0031
**PVR vs. No PVR**	**CTSL**	1.65	0.004
**PVR vs. No PVR**	**AHSG**	1.65	0.01
**PVR vs. No PVR**	**CHL1**	1.66	0.0072
**PVR vs. No PVR**	**ENO2**	1.67	0.0071
**PVR vs. No PVR**	**C8G**	1.67	0.0019
**PVR vs. No PVR**	**COL11A1**	1.68	0.011
**PVR vs. No PVR**	**CD59**	1.69	0.0023
**PVR vs. No PVR**	**ATP6AP1**	1.70	0.0024
**PVR vs. No PVR**	**PIK3IP1**	1.72	0.0027
**PVR vs. No PVR**	**PAPPA2**	1.72	0.0056
**PVR vs. No PVR**	**CLEC3B**	1.73	0.0072
**PVR vs. No PVR**	**C1QB**	1.74	0.0021
**PVR vs. No PVR**	**RNASE1**	1.75	0.0048
**PVR vs. No PVR**	**IGHV6-1**	1.75	0.002
**PVR vs. No PVR**	**TNR**	1.76	0.0037
**PVR vs. No PVR**	**FAM3C**	1.77	0.0021
**PVR vs. No PVR**	**PCSK1N**	1.78	0.0012
**PVR vs. No PVR**	**MMP2**	1.79	0.0024
**PVR vs. No PVR**	**APOH**	1.80	0.004
**PVR vs. No PVR**	**RTBDN**	1.82	0.0013
**PVR vs. No PVR**	**SERPINI1**	1.83	0.00038
**PVR vs. No PVR**	**CNDP1**	1.83	0.00042
**PVR vs. No PVR**	**RNASET2**	1.83	0.0024
**PVR vs. No PVR**	**GPX3**	1.84	0.0016
**PVR vs. No PVR**	**B2M**	1.84	0.0068
**PVR vs. No PVR**	**PLD3**	1.87	0.0024
**PVR vs. No PVR**	**FUCA2**	1.87	0.0025
**PVR vs. No PVR**	**IMPG1**	1.89	0.014
**PVR vs. No PVR**	**NRXN3**	1.89	0.00065
**PVR vs. No PVR**	**TPP1**	1.89	0.00081
**PVR vs. No PVR**	**NCAN**	1.89	0.0011

Differentially Expressed proteins			
**Comparison**	**SYMBOL**	**logFold**	**p-value**
**PVR vs. No PVR**	**SERPINA4**	1.90	0.0037
**PVR vs. No PVR**	**SOD3**	1.90	0.0048
**PVR vs. No PVR**	**C8A**	1.90	0.00072
**PVR vs. No PVR**	**LRP1**	1.94	0.0013
**PVR vs. No PVR**	**PON1**	1.94	0.00098
**PVR vs. No PVR**	**APOD**	1.95	0.007
**PVR vs. No PVR**	**ECM1**	1.95	0.0018
**PVR vs. No PVR**	**FBLN1**	1.97	0.00089
**PVR vs. No PVR**	**TPI1**	1.99	0.0011
**PVR vs. No PVR**	**APOA4**	2.00	0.0042
**PVR vs. No PVR**	**MACF1**	2.01	0.00037
**PVR vs. No PVR**	**HSPA5**	2.01	0.00061
**PVR vs. No PVR**	**FSTL4**	2.01	0.0021
**PVR vs. No PVR**	**GOLM1**	2.01	0.0019
**PVR vs. No PVR**	**KNG1**	2.01	0.00097
**PVR vs. No PVR**	**COL6A1**	2.03	0.00014
**PVR vs. No PVR**	**CRTAC1**	2.03	0.00049
**PVR vs. No PVR**	**CPQ**	2.03	0.0006
**PVR vs. No PVR**	**PLG**	2.06	0.0011
**PVR vs. No PVR**	**C2**	2.07	0.00056
**PVR vs. No PVR**	**APP**	2.07	0.00037
**PVR vs. No PVR**	**PDE6A**	2.07	0.00061
**PVR vs. No PVR**	**IGFBP7**	2.11	0.0011
**PVR vs. No PVR**	**ATRN**	2.11	0.0001
**PVR vs. No PVR**	**SPOCK1**	2.13	0.00059
**PVR vs. No PVR**	**CFHR2**	2.14	0.0022
**PVR vs. No PVR**	**APOE**	2.16	0.00048
**PVR vs. No PVR**	**AMBP**	2.16	0.00047
**PVR vs. No PVR**	**LSAMP**	2.17	0.00052
**PVR vs. No PVR**	**IMPG2**	2.19	0.0023
**PVR vs. No PVR**	**F2**	2.24	0.00026
**PVR vs. No PVR**	**WIF1**	2.24	0.000056
**PVR vs. No PVR**	**CDH2**	2.27	0.0015
**PVR vs. No PVR**	**COL18A1**	2.28	0.00017
**PVR vs. No PVR**	**FRZB**	2.31	0.00012
**PVR vs. No PVR**	**CTSD**	2.35	0.0003
**PVR vs. No PVR**	**GNB1**	2.35	0.00014
**PVR vs. No PVR**	**LRP2**	2.35	0.00019
**PVR vs. No PVR**	**GNAT1**	2.43	0.000066

Differentially Expressed proteins			
**Comparison**	**SYMBOL**	**logFold**	**p-value**
**PVR vs. No PVR**	**PTGDS**	2.43	0.00014
**PVR vs. No PVR**	**RS1**	2.46	0.00005
**PVR vs. No PVR**	**CPAMD8**	2.47	0.00036
**PVR vs. No PVR**	**NRCAM**	2.51	0.000011
**PVR vs. No PVR**	**SPON1**	2.58	5.4E-06
**PVR vs. No PVR**	**RBP3**	2.59	0.000042
**PVR vs. No PVR**	**CPVL**	2.60	0.00018
**PVR vs. No PVR**	**NPC2**	2.63	0.000047
**PVR vs. No PVR**	**RHO**	2.67	0.000012
**PVR vs. No PVR**	**B4GAT1**	2.68	7.7E-06
**PVR vs. No PVR**	**ABI3BP**	2.70	0.000012
**PVR vs. No PVR**	**CPE**	2.70	0.000016
**PVR vs. No PVR**	**ACTB**	2.71	0.00011
**PVR vs. No PVR**	**C4B**	2.74	0.0067
**PVR vs. No PVR**	**CLU**	2.79	0.000011
**PVR vs. No PVR**	**VCAN**	2.81	0.000012
**PVR vs. No PVR**	**CFH**	2.87	7.6E-06
**PVR vs. No PVR**	**CDHR1**	2.93	0.00023
**PVR vs. No PVR**	**EFEMP1**	2.95	2.9E-06
**PVR vs. No PVR**	**ENPP2**	2.97	0.000067
**PVR vs. No PVR**	**CFI**	3.08	1.6E-06
**PVR vs. No PVR**	**PKM**	3.16	7.1E-06
**PVR vs. No PVR**	**HSPG2**	3.25	3.8E-07
**PVR vs. No PVR**	**COL2A1**	3.29	1.7E-06
**PVR vs. No PVR**	**FSTL5**	3.32	3.8E-07
**PVR vs. No PVR**	**IGLL5**	3.39	3.7E-06
**PVR vs. No PVR**	**SEMA7A**	3.41	2.6E-06
**PVR vs. No PVR**	**CLSTN1**	3.46	7.5E-08
**PVR vs. No PVR**	**SPP1**	3.59	1.3E-06
**PVR vs. No PVR**	**APLP2**	4.05	7.4E-09
**PVR vs. No PVR**	**CST3**	4.07	4.6E-08
**PVR vs. No PVR**	**DKK3**	4.11	1.1E-07

Supplementary Table S3

SYMBOL	No PVR 1	No PVR2	Mld PVR1	Mld PVR2	Svr PVR1	Svr PR2	Svr PVR3	Svr PVR4	Svr PVR5
CFH	13.413	13.548	9.023	8.995	12.674	13.141	13.6	8.623	12.32
F2	12.808	12.268	9.023	8.995	12.403	13.018	12.815	8.623	11.098
KNG1	12.532	12.268	9.023	8.995	12.189	13.081	13.191	8.623	11.628
PLG	12.372	11.963	9.023	8.995	12.189	12.736	13.238	8.623	10.25
AMBP	12.676	11.335	9.023	8.995	10.715	11.919	12.988	8.623	10.25
TF	15.597	15.391	13.667	13.243	14.803	15.089	15.035	13.016	14.498
SERPING1	13.71	13.49	11.831	11.317	12.903	13.457	12.546	11.793	12.572
EFEMP1	12.676	13.005	9.023	8.995	11.246	12.656	12.39	8.623	10.25
CTSD	12.676	13.233	9.023	10.58	11.937	13.901	12.933	10.208	10.25
APOE	13.642	14.215	12.724	11.317	12.499	13.457	13.093	8.623	10.735
CPAMD8	11.744	11.576	9.023	8.995	11.005	11.433	8.845	8.623	7.928
ENPP2	12.191	12.734	9.023	8.995	11.452	12.953	12.546	8.623	7.928
C2	11.454	11.046	9.023	8.995	9.867	11.433	11.508	8.623	7.928
PON1	11.091	11.046	9.023	8.995	9.867	10.696	10.961	8.623	7.928
CFI	12.372	12.92	9.023	8.995	10.353	12.656	11.653	8.623	9.513
CLU	14.971	15.569	12.193	12.165	13.428	15.261	14.226	10.945	11.628
C1QB	10.606	11.046	9.023	8.995	9.867	10.986	10.068	8.623	7.928
RBP3	15.784	15.152	13.881	13.387	13.1	15.353	13.45	8.623	11.098
GPX3	13.238	12.92	11.831	11.802	11.005	13.081	11.653	8.623	10.25
DKK3	13.142	13.548	9.023	8.995	10.353	12.571	10.72	8.623	7.928
C8G	10.606	10.683	9.023	8.995	9.13	10.696	10.068	8.623	7.928
ATRN	11.454	10.683	9.023	8.995	9.13	10.334	10.068	8.623	7.928
C8A	11.454	10.198	9.023	8.995	9.13	10.334	9.582	8.623	7.928
FBLN1	11.744	10.198	9.023	8.995	9.13	11.227	10.068	8.623	7.928
IGLL5	12.372	12.268	9.023	8.995	7.545	11.614	12.215	8.623	7.928
ACTB	11.091	11.963	9.023	8.995	7.545	11.614	10.72	8.623	7.928
PTGDS	14.694	14.721	12.93	12.165	11.246	14.765	13.89	12.083	11.098
IGFBP7	11.091	10.683	9.023	8.995	7.545	10.986	10.43	8.623	7.928
SPP1	12.532	12.399	9.023	8.995	7.545	11.614	11.348	8.623	7.928
CDHR1	11.454	11.963	9.023	8.995	7.545	11.774	10.068	8.623	7.928
CLSTN1	12.928	11.963	9.023	8.995	9.13	11.227	9.582	8.623	7.928
FAM3C	9.869	11.046	9.023	8.995	7.545	10.334	9.582	8.623	7.928
CST3	13.039	12.734	9.023	8.995	7.545	11.614	10.43	8.623	7.928
NPC2	11.744	11.046	9.023	8.995	7.545	10.696	10.43	8.623	7.928
B4GAT1	11.454	11.335	9.023	8.995	7.545	10.334	10.068	8.623	7.928
TPI1	10.606	10.683	9.023	8.995	7.545	9.848	9.582	8.623	7.928
ECM1	10.606	10.683	9.023	8.995	7.545	9.848	10.068	8.623	7.928

**PKM**	12.191	11.576	9.023	8.995	7.545	9.848	10.43	8.623	7.928
**PCSK1N**	10.606	10.198	9.023	8.995	7.545	9.111	9.582	8.623	7.928
**SPON1**	10.606	11.782	9.023	8.995	7.545	9.111	9.582	8.623	7.928
**GOLM1**	10.606	10.683	9.023	8.995	7.545	10.334	8.845	8.623	7.928
**RTBDN**	9.869	11.046	9.023	8.995	7.545	10.334	8.845	8.623	7.928
**COL6A1**	10.606	10.683	9.023	8.995	7.545	9.848	8.845	8.623	7.928
**CRTAC1**	10.606	10.683	9.023	8.995	7.545	9.848	8.845	8.623	7.928
**CPQ**	10.606	10.683	9.023	8.995	7.545	9.848	8.845	8.623	7.928
**FSTL5**	11.985	11.963	9.023	8.995	7.545	10.696	8.845	8.623	7.928
**SEMA7A**	12.191	11.963	9.023	8.995	7.545	10.986	8.845	8.623	7.928
**COL18A1**	11.091	10.683	9.023	8.995	7.545	9.848	8.845	8.623	7.928
**CPVL**	11.091	11.335	9.023	8.995	7.545	9.848	8.845	8.623	7.928
**SERPINI1**	10.606	10.198	9.023	8.995	7.545	9.111	8.845	8.623	7.928
**CNDP1**	10.606	10.198	9.023	8.995	7.545	9.111	8.845	8.623	7.928
**FRZB**	11.091	10.683	9.023	8.995	7.545	9.111	8.845	8.623	7.928
**APP**	11.091	10.198	9.023	8.995	7.545	9.111	8.845	8.623	7.928
**CDH2**	10.606	11.046	9.023	8.995	7.545	10.696	7.26	8.623	7.928
**NRXN3**	10.606	10.198	9.023	8.995	7.545	9.848	7.26	8.623	7.928
**TPP1**	10.606	10.198	9.023	8.995	7.545	9.848	7.26	8.623	7.928
**NRCAM**	11.454	10.683	9.023	8.995	7.545	10.696	7.26	8.623	7.928
**GNB1**	11.091	10.683	9.023	8.995	7.545	10.334	7.26	8.623	7.928
**LRP2**	11.091	10.683	9.023	8.995	7.545	10.334	7.26	8.623	7.928
**COL2A1**	11.744	11.782	9.023	8.995	7.545	9.111	7.26	8.623	7.928
**APLP2**	12.372	12.734	9.023	8.995	7.545	9.848	7.26	8.623	7.928
**HSPG2**	11.744	11.782	9.023	8.995	7.545	9.848	7.26	8.623	7.928
**ABI3BP**	11.091	11.335	9.023	8.995	7.545	9.848	7.26	8.623	7.928
**CPE**	11.091	11.335	9.023	8.995	7.545	9.848	7.26	8.623	7.928
**LRP1**	9.869	11.046	9.023	8.995	7.545	9.848	7.26	8.623	7.928
**IGHV6-1**	9.869	10.683	9.023	8.995	7.545	9.848	7.26	8.623	7.928
**SIAE**	9.869	10.198	9.023	8.995	7.545	9.111	7.26	8.623	7.928
**SPOCK1**	11.091	10.198	9.023	8.995	7.545	9.848	7.26	8.623	7.928
**VCAN**	11.985	10.683	9.023	8.995	7.545	9.848	7.26	8.623	7.928
**RS1**	11.744	10.198	9.023	8.995	7.545	9.848	7.26	8.623	7.928
**LSAMP**	11.091	10.198	9.023	8.995	7.545	9.111	7.26	8.623	7.928
**RHO**	11.091	11.046	9.023	8.995	7.545	7.526	7.26	8.623	7.928
**GNAT1**	11.454	10.198	9.023	8.995	7.545	7.526	7.26	8.623	7.928
**WIF1**	10.606	10.683	9.023	8.995	7.545	7.526	7.26	8.623	7.928
**MACF1**	10.606	10.198	9.023	8.995	7.545	7.526	7.26	8.623	7.928
**HSPA5**	10.606	10.198	9.023	8.995	7.545	7.526	7.26	8.623	7.928
**FSTL4**	10.606	10.198	9.023	8.995	7.545	7.526	7.26	8.623	7.928
**PDE6A**	11.454	9.461	9.023	8.995	7.545	7.526	7.26	8.623	7.928
**NCAN**	11.091	9.461	9.023	8.995	7.545	7.526	7.26	8.623	7.928

**QSOX1**	10.606	9.461	9.023	8.995	7.545	7.526	7.26	8.623	7.928
**KRT73**	8.284	7.876	11.831	12.454	7.545	7.526	7.26	12.324	7.928
**KRT17**	8.284	7.876	13.778	13.243	7.545	7.526	7.26	13.378	7.928
**KRT25**	8.284	7.876	13.111	13.387	7.545	7.526	7.26	12.871	7.928
**KRT6B**	8.284	7.876	14.856	14.828	12.189	7.526	8.845	14.927	7.928
**KRT9**	11.744	10.198	15.71	16.028	12.674	9.848	7.26	15.566	14.498
**KRT14**	8.284	11.576	14.309	14.28	11.005	10.696	9.582	14.646	12.882
**KRT78**	8.284	7.876	11.831	11.317	7.545	7.526	7.26	11.431	10.25
**KRT6A**	8.284	7.876	14.578	15.145	7.545	7.526	7.26	14.813	13.656
**KRT16**	8.284	7.876	14.152	14.124	7.545	7.526	7.26	14.405	12.683
**KRT10**	12.532	12.63	16.601	16.702	13.849	10.986	10.068	16.705	16.103
**KRT1**	12.372	11.782	16.771	16.92	13.849	11.433	10.43	16.572	15.622
**KRT2**	12.191	11.963	16.281	16.511	13.037	11.614	10.72	16.289	15.185
**KRT77**	8.284	7.876	13.547	13.949	9.867	7.526	7.26	13.267	12.32
**KRT71**	8.284	7.876	12.93	13.518	9.867	7.526	7.26	12.083	11.098
**KRT5**	8.284	7.876	14.638	15.061	11.633	7.526	7.26	14.89	13.354
**IGKV4-1**	8.284	7.876	10.608	10.58	10.715	9.111	9.582	10.208	11.098
**IGHG2**	8.284	7.876	13.271	13.75	14.511	12.656	13.191	13.577	15.088
**IGLL1**	8.284	7.876	12.724	8.995	13.378	7.526	7.26	13.016	13.354
